# Supplementary material for: Dynamic control of endogenous metabolism with combinatorial logic circuits
Source: Mol Syst Biol. 2018 Nov 29;14(11):e8605. doi: 10.15252/msb.20188605 (PMC6263354; doi:10.15252/msb.20188605)
Supplement: Supplementary file 1 — Appendix [file MSB-14-e8605-s001.docx]

Appendix

**Dynamic control of endogenous metabolism with combinatorial logic circuits**

Moser, F., Espah Borujeni, A., Ghodasara, A., Cameron, E. D., Park, Y.-J. and C.A. Voigt.

Table of Contents

1. Appendix Figures

**Appendix Figure S1: Responses of select *E. coli* promoters to glucose and oxygen.**

**Appendix Figure S2: Cytometry distributions corresponding to sensor responses.**

**Appendix Figure S3: Glucose and acetate sensor responses over time.**

**Appendix Figure S4: Effect of acetate pathway deletions on acetate sensor response.**

**Appendix Figure S5: Fluorescence values corresponding to the sensor orthogonality grid.**

**Appendix Figure S6: Relationship between cell density (OD_600_) and dissolved oxygen (DO).**

**Appendix Figure S7: Cytometry distributions corresponding to sensor responses during growth in shake flasks.**

**Appendix Figure S8: Simulations of circuit dynamics.**

**Appendix Figure S9: Cytometry distributions corresponding to 2-input logic gates.**

**Appendix Figure S10: Cytometry distributions corresponding to 3-input logic circuits.**

**Appendix Figure S11: Cytometry distributions corresponding to RFP repression by CRISPRi, sRNA, and *mf*-LON.**

**Appendix Figure S12: Tuning *mf*-LON expression to reduce toxicity.**

**Appendix Figure S13: Fusion of sgRNA and CDS transcripts generates functional sgRNA and protease.**

**Appendix Figure S14: SuMMV degradation tag insertion into PoxB structure.**

**Appendix Figure S15: Effect of *pta* and *poxB* deletions on the dynamics of acetate production.**

**Appendix Figure S16: Growth curves of circuit strains.**

**Appendix Figure S17: Plasmid Maps**

**Appendix Table S1: Growth rates of acetate pathway mutants**

**Appendix Table S2: Genetic parts used in this work**

1. References

**I. Appendix Figures**

**
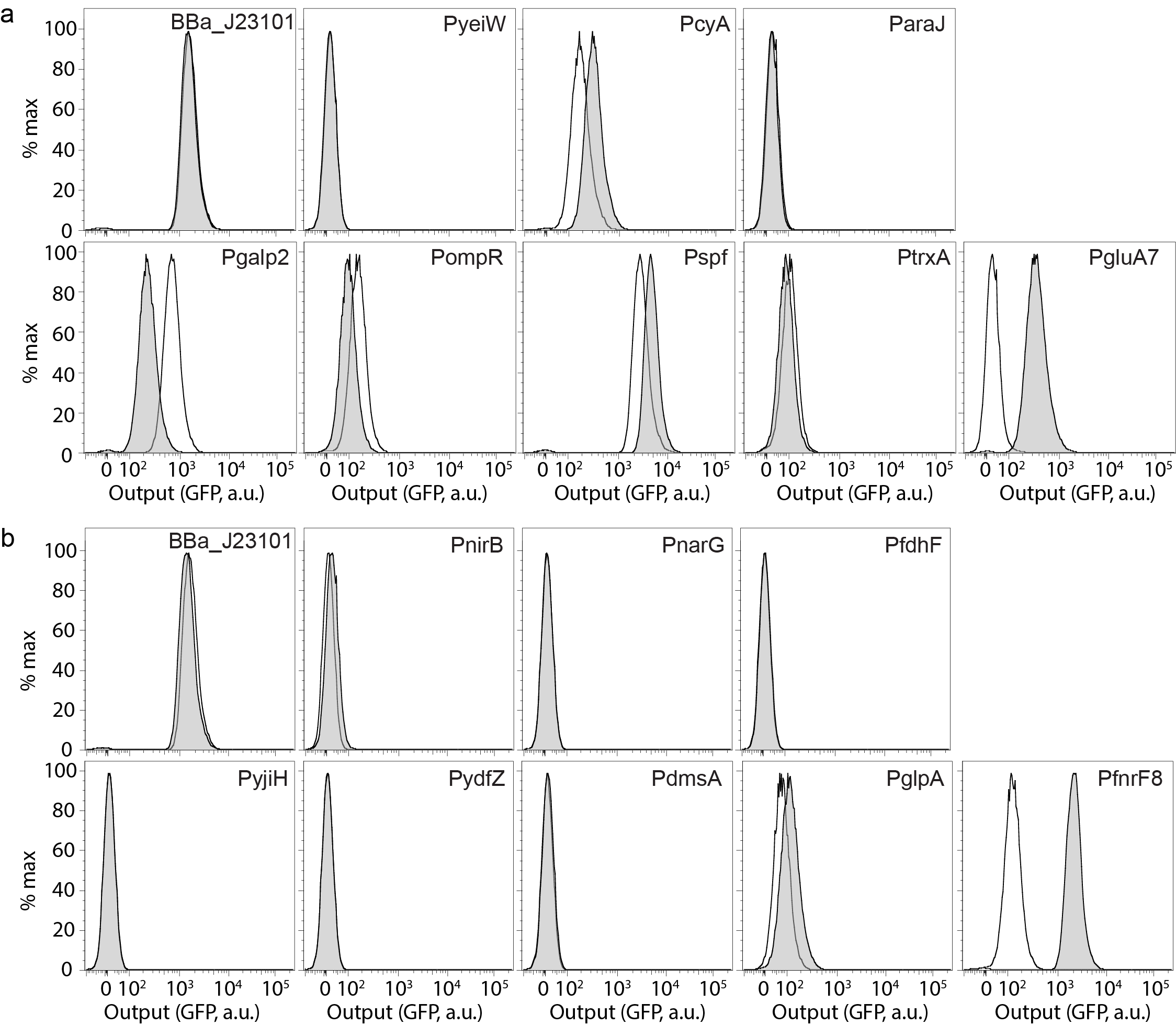
**

**Appendix Figure S1: Responses of select *E. coli* promoters to glucose and oxygen.** Native *E. coli* MG1655 promoter sequences (Appendix Table S2) were cloned into pFM438 in place of the BBa_J23101 promoter. Each resulting plasmid was transformed into MG1655*ΔglnL* and the resulting strains’ GFP response was tested alongside the synthetic sensor promoters PgluA7 and PfnrF8 (Methods). **(a)** Cytometry distributions of GFP fluorescence of native *E. coli* CRP-regulated promoters and the synthetic promoters BBa_J23101 and PgluA7 to growth in minimal media containing 0.4% glycerol (unshaded) or 0.4% glucose (shaded) after 6 hours. **(b)** Cytometry GFP distribution of native *E. coli* FNR-regulated promoters and the synthetic promoters BBa_J23101 and PfnrF8 after growth on minimal media containing 0.4% glycerol in either aerobic (unshaded) or anaerobic (shaded) conditions after 6 hours.

**
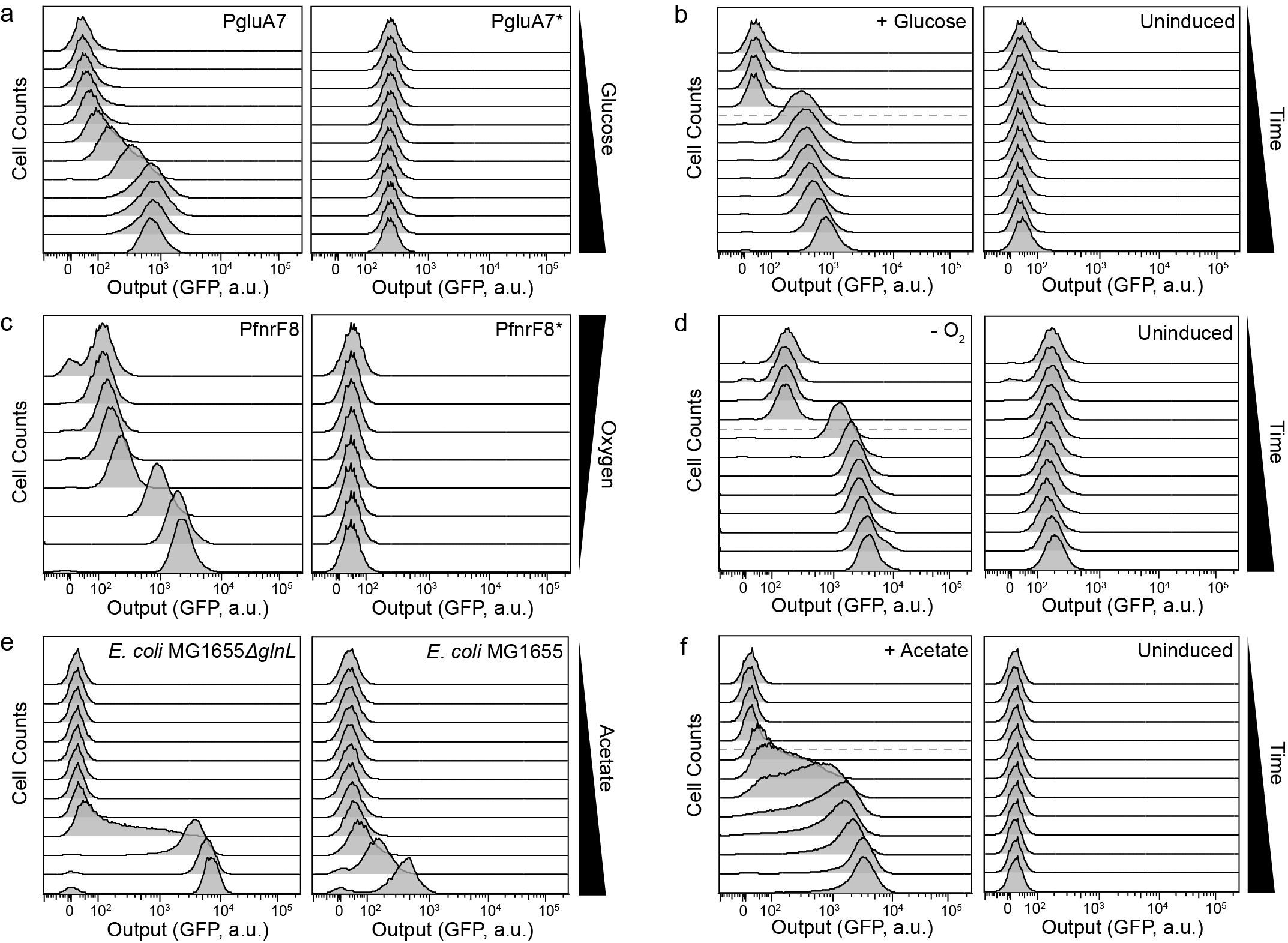
**

**Appendix Figure S2: Cytometry distributions corresponding to sensor responses.** Cytometry distributions for the glucose, oxygen, and acetate sensors shown here corresponding to data from Figure 1f, 1g, and 1h, respectively. **(a)** Response of the glucose sensor (PgluA7) and the glucose sensor with the CRP operator randomized (PgluA7*). Glucose concentrations from top to bottom are as follows: 0, 0.0015%, 0.0031%, 0.0063%, 0.0125%, 0.0250%, 0.05%, 0.1%, 0.2%, 0.4%, 0.8%, and 1.6%. **(b)** Dynamic response of the glucose sensor (PgluA7) either induced with 0.8% glucose (+Glucose) or grown only on 0.8% glycerol (Uninduced). **(c)** Response functions of the oxygen sensor (PfnrF8) and the oxygen sensor with the FNR operator randomized (PfnrF8*). **(d)** Dynamic response of the oxygen sensor (PfnrF8) either induced with anaerobic growth (-O_2_) or grown aerobically (Uninduced). **(e)** Response function of the acetate sensor (PglnAP2s) in *E. coli* MG1655 *ΔglnL* and unmodified *E. coli* MG1655. Acetate concentrations from top to bottom are as follows: 0, 0.098, 0.195, 0.390, 0.781, 1.562, 3.125, 6.25, 12.5, 25, 50, and 100 mM. **(f)** Dynamic response of the acetate sensor (PglnAP2s) either induced with 30 mM acetate (+Acetate) or grown without acetate (Uninduced). For dynamic responses, all time intervals are 1 hour and the dashed line indicates the time of induction.


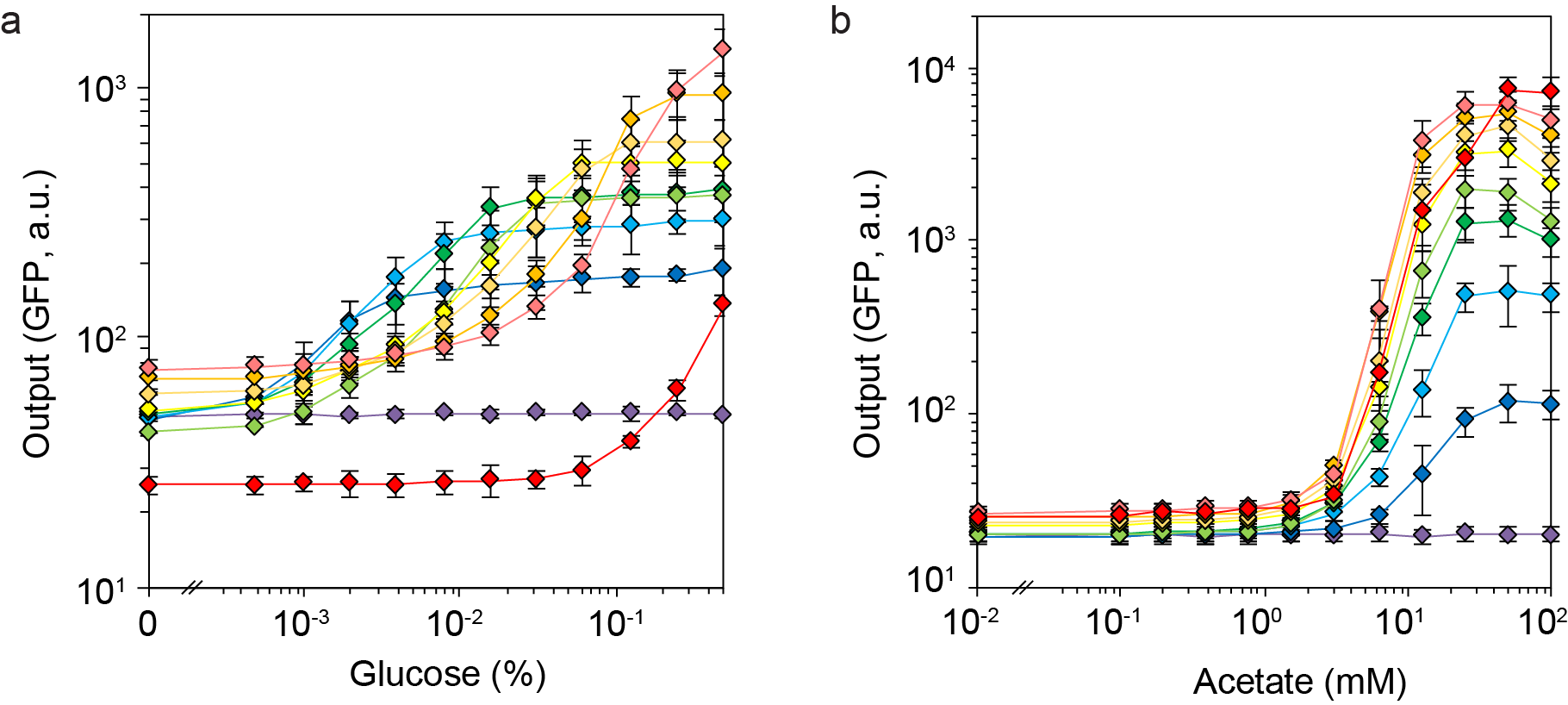


**Appendix Figure S3: Glucose and acetate sensor responses over time.** Shown are the complete responses of **(a)** the glucose sensor (PgluA7) and **(b)** the acetate sensor (PglnAP2s) over time. Note the change in the glucose response as glucose is consumed by the culture over time. Cultures were grown in MM containing 0.4% glycerol and were induced immediately after sampling at time 0. The coloration of the time progression is: 0 hours (purple), 1 hour (light blue), 2 hours (dark blue), 3 hours (light green), 4 hours (dark green), 5 hours (yellow), 6 hours (darker yellow), 7 hours (light orange), 8 hours (orange), 9 hours (light red), 18 hours (dark red). Error bars are one standard deviation of three experiments performed on different days.


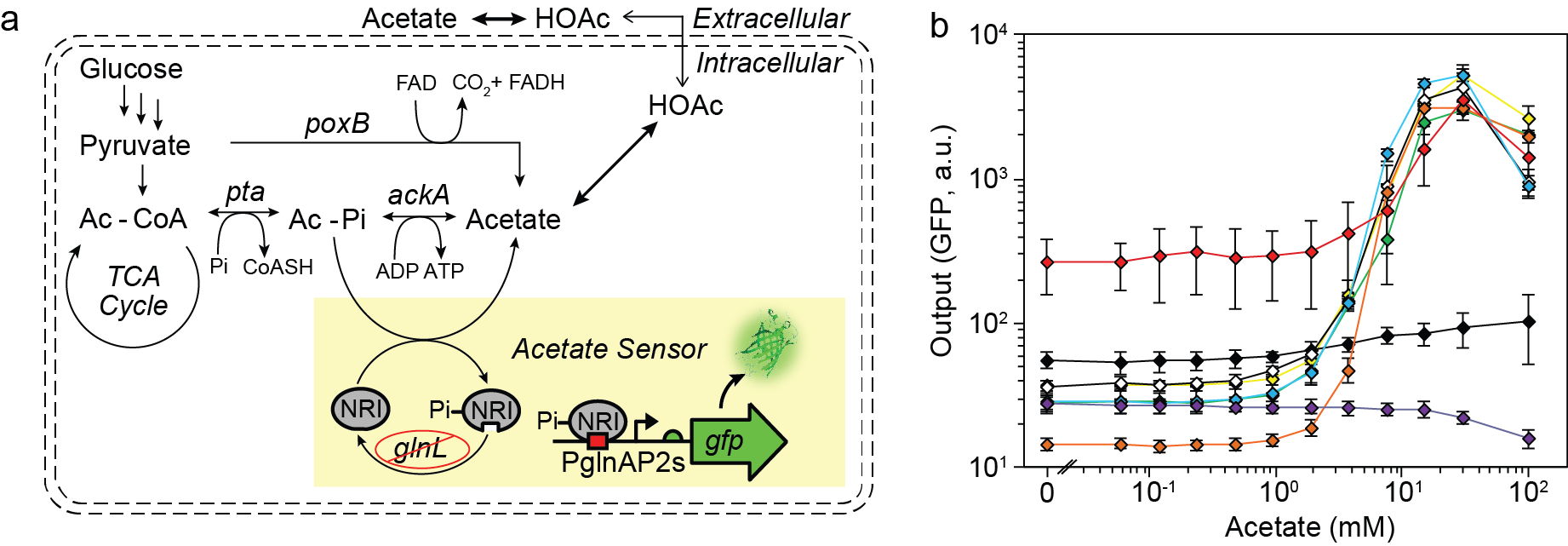


**Appendix Figure S4: Effect of acetate pathway deletions on acetate sensor response. (a)** Diagram of the metabolic pathways to acetate and their connection to the acetate sensor in *Escherichia coli*. **(b)** Induction of the acetate sensor in knockout strains of the *E. coli* acetate pathway. The following strains all contained the unmodified acetate sensor promoter (PglnAP2): *E. coli* MG1655 (black), *ΔglnL* (white), *ΔglnLΔackA* (red), *ΔglnLΔpta* (blue), *Δ*glnL*Δ*pta*Δ*poxB (green), *Δ*glnL*Δ*poxB (yellow), and *Δ*glnL*Δ*ackA*Δ*pta (purple), respectively. The *ΔglnL* strain containing the engineered acetate sensor promoter (PglnAP2s) plasmid pFM704 is shown in orange. Error bars are one standard deviation of three experiments performed on different days.


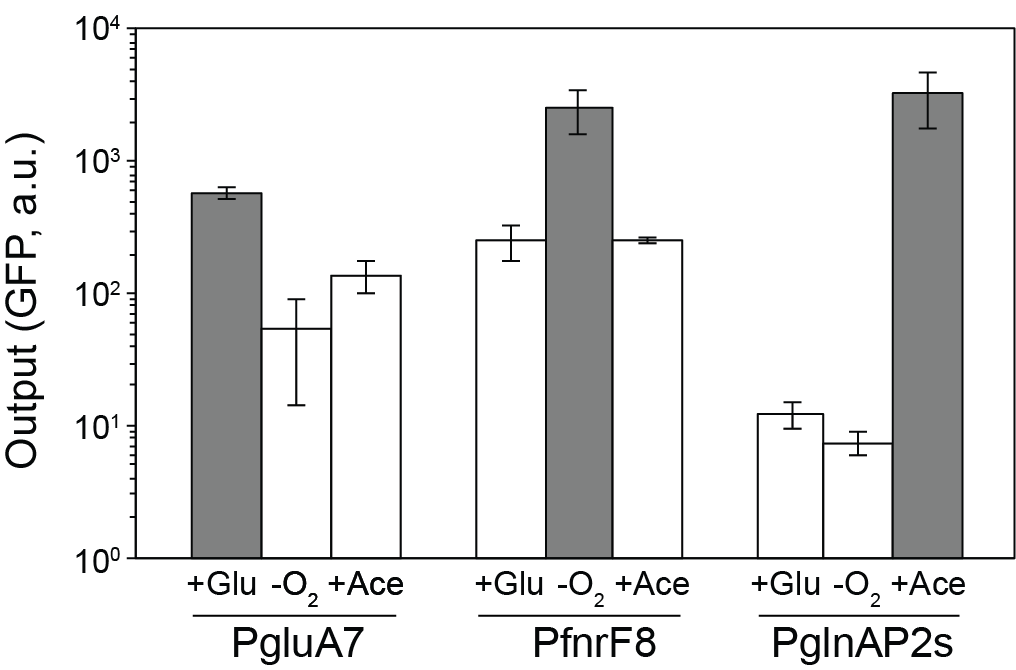


**Appendix Figure S5: Fluorescence values corresponding to the sensor orthogonality grid.** Shown are the fluorescence values corresponding to Figure 1e. The GFP fluorescence values of each culture were measured by cytometry after 7 hours of growth in either 0.8% glucose (+Glu), anaerobic conditions (-O_2_), 20 mM acetate (+Ace). Shown are median fluorescence values. Cognate induction is shaded grey. Error bars are one standard deviation of three experiments performed on different days.


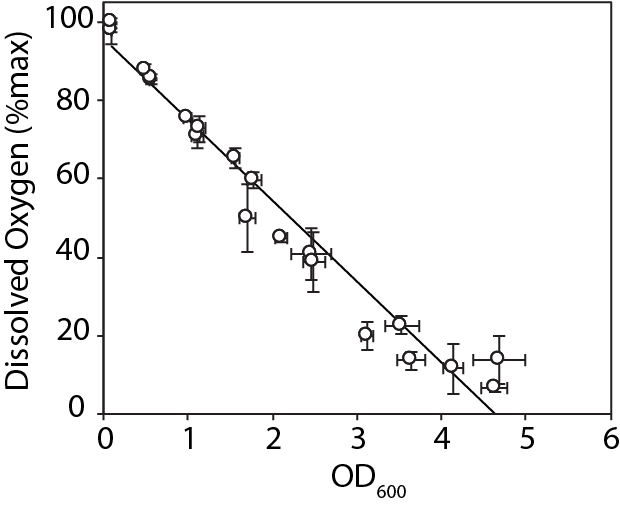


**Appendix Figure S6: Relationship between culture density (OD_600_) and dissolved oxygen (DO).** Shown is the relationship between the culture density (OD_600_) of an *E. coli* MG1655 culture growing in 30 ml of MM containing 1.6% glucose and the measured dissolved oxygen (DO). The DO is calibrated to a flask of identical media at 37°C shaken vigorously for 5 minutes and was measured as described (Materials & Methods). The black line is the linear regression. The culture was grown in 250 ml un-baffled shake flasks at 250 RPM with a throw length of 1 inch. Here, 100% dissolved oxygen was measured to be 213 µmol/L. All measurements of OD_600_ above 5 all showed DO values below the sensitivity threshold of the DO probe and were omitted. Each point represents the average for three biological replicates and error bars represent one standard deviation of three biological replicates.


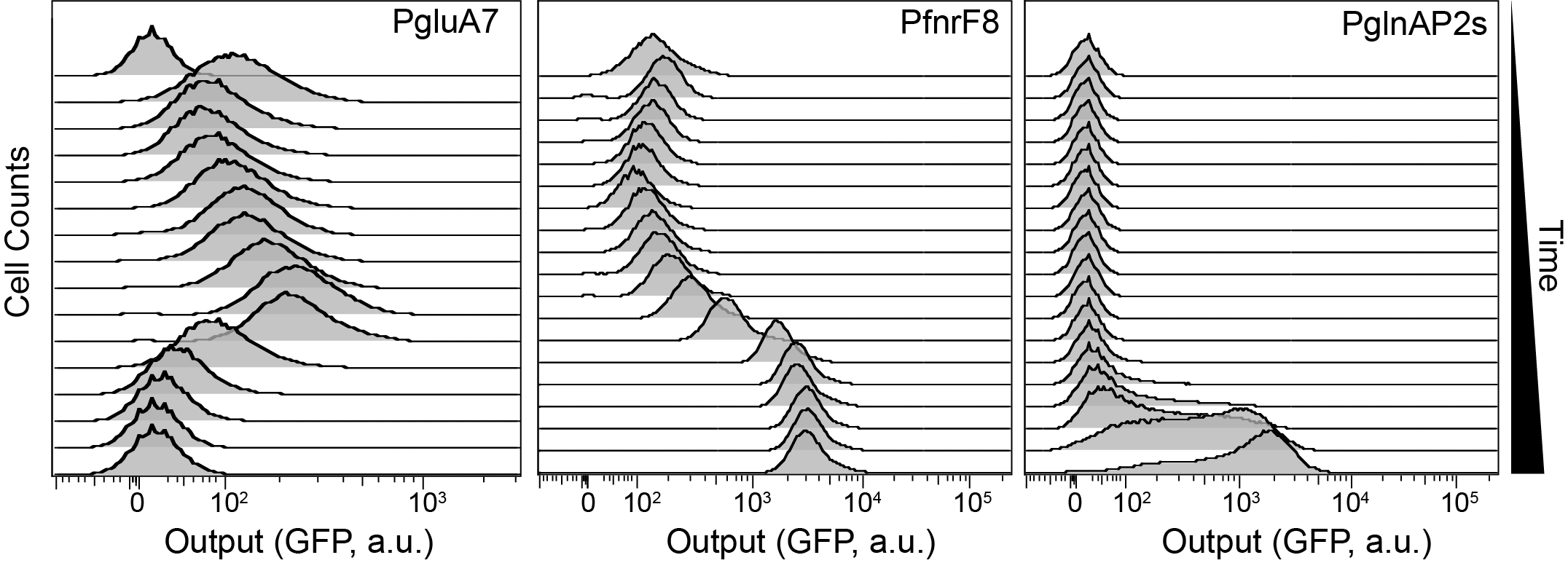


**Appendix Figure S7: Cytometry distributions corresponding to sensor responses during growth in shake flasks.** Shown are the cytometry data to Figure 2a,b,c. Time progressions indicated on the right are in intervals of 1 hour.


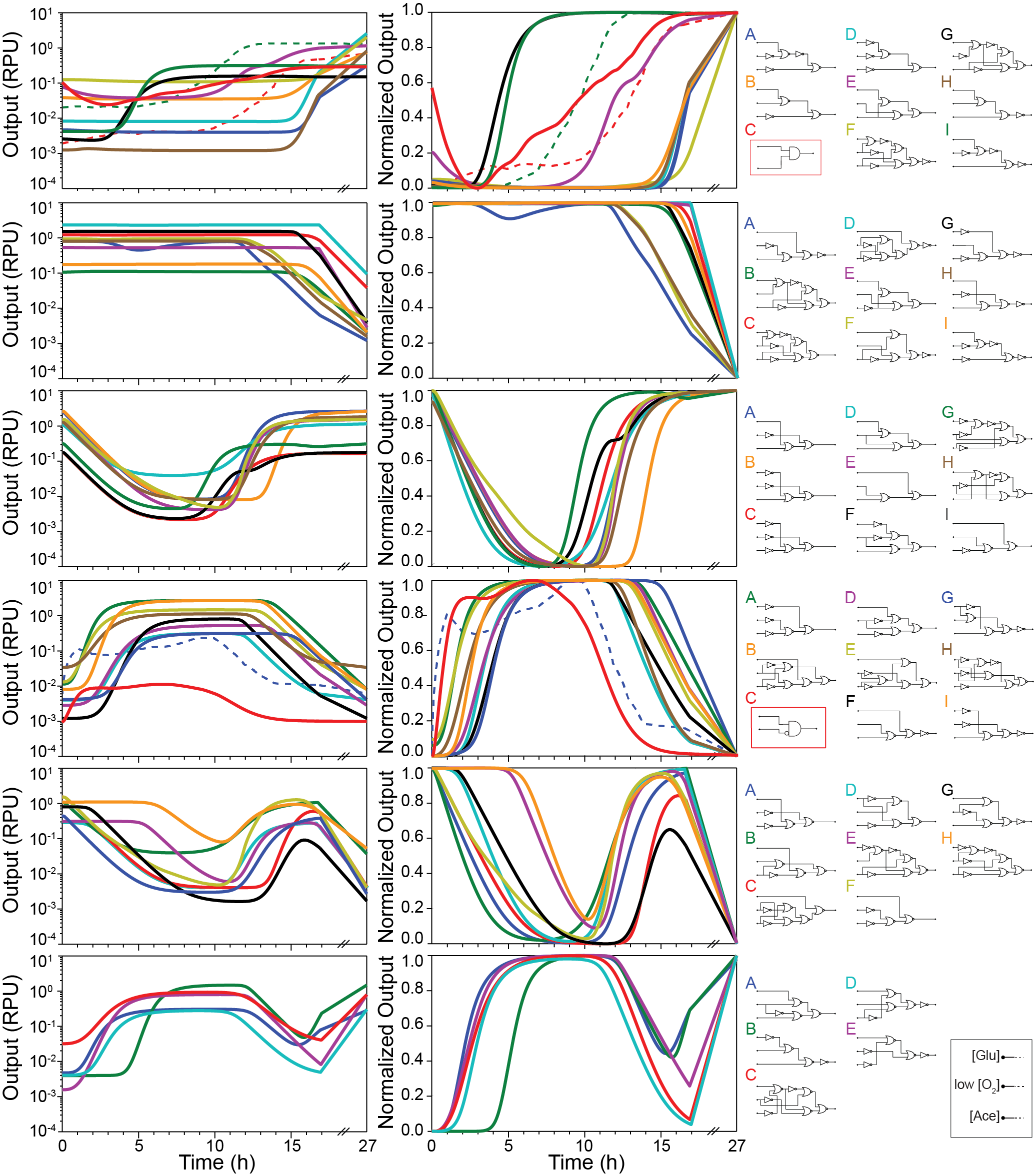


**Supplementary Figure S8: Simulations of circuit dynamics.** Cello software was used to design 124 3-input, 1-output circuits that convert the input signals (glucose, oxygen, and acetate) to output signal (RPU). The dynamic response of the output signal was modeled for each circuit during a 27-hour shake flask growth on glucose based on the time-dependent response of the input sensors (glucose, oxygen, acetate). Plots show the six most common responses from a selection of the 124 circuits, including (from top to bottom): turn ON, turn OFF, negative pulse, positive pulse, wave, and inverse wave. Colors of output curves correspond to the colors of the letters marking circuit diagrams on the right. The circuits shown in red boxes are the circuits from Figure 2e. Top, middle, and bottom input lines correspond to glucose, low oxygen, and acetate inputs, respectively. The right column of plots show the normalized output signal for each circuit in the adjacent left plots, which was calculated using the formula: $\frac{\log_{10} (X\left( t \right)/X_{min})}{\log_{10} {(X}_{max}/X_{min})}$, where X(t) is the circuit output at time t.


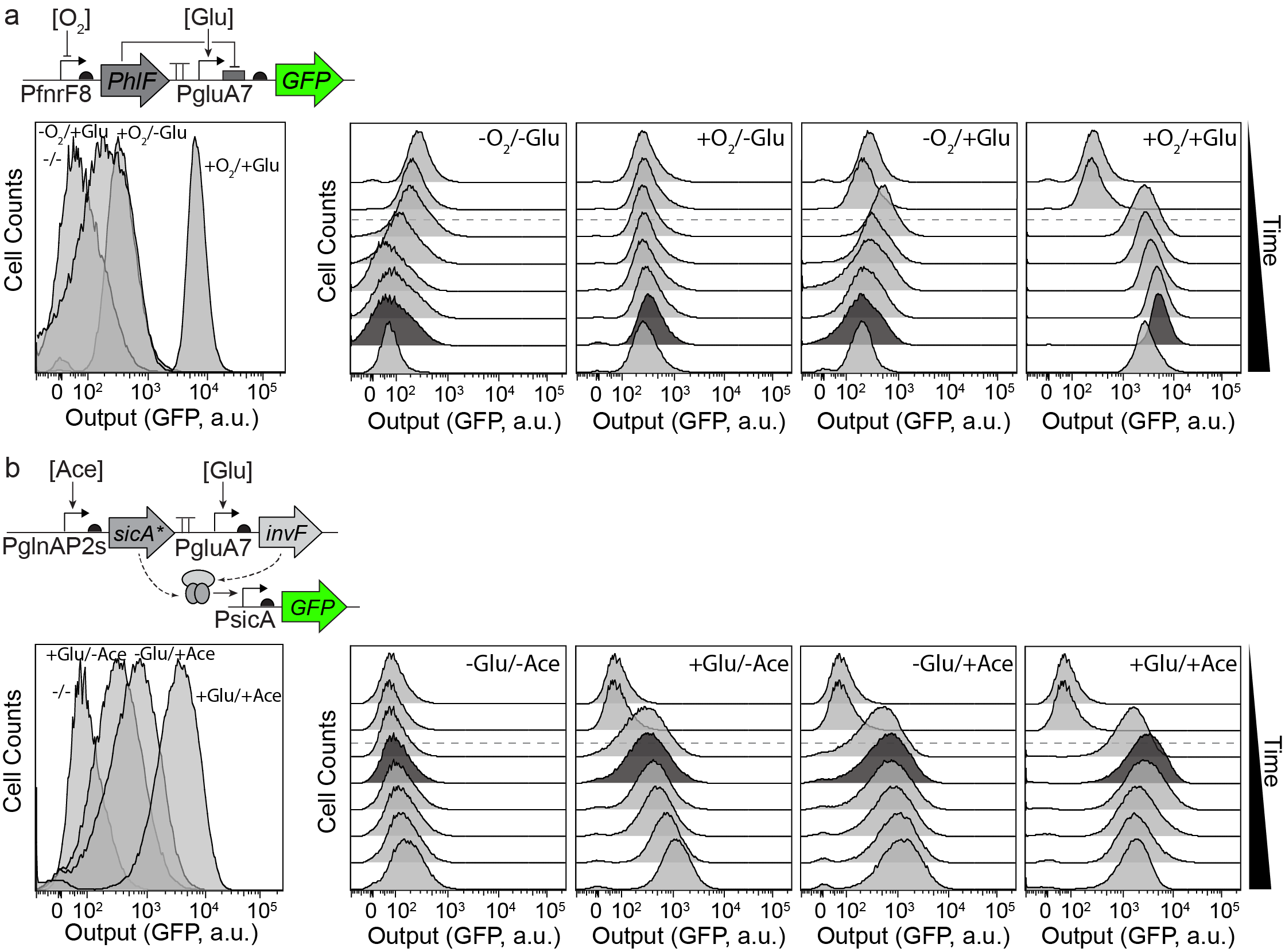


**Appendix Figure S9: Cytometry distributions corresponding to 2-input logic gates.** In this work, sensors were combined into 2-input logic gates by one of two mechanisms: the insertion of a PhlF operator downstream of the +1 site of a promoter, or the expression of a 2-component activator (sicA/invF) system. **(a)** PfnrF8 is used to drive PhlF expression to regulate the output of PgluA7 to compose a low-oxygen/glucose AND gate. **(b)** PglnAP2s and PgluA7 are used to drive sicA and invF, respectively, to create a single layer of AND logic. Below each genetic diagram are the cytometry distributions of the gate in each inducing condition. To the right are shown the dynamic responses of the gates in each condition at time intervals of 1 hour. Each culture was grown in MM + 0.4% glycerol (methods). Dashed lines indicate the time of induction with 30 mM acetate, 0.8% glucose, or anaerobic growth conditions. Darkened distributions in the dynamic responses correspond to the distributions shown at the left.

**
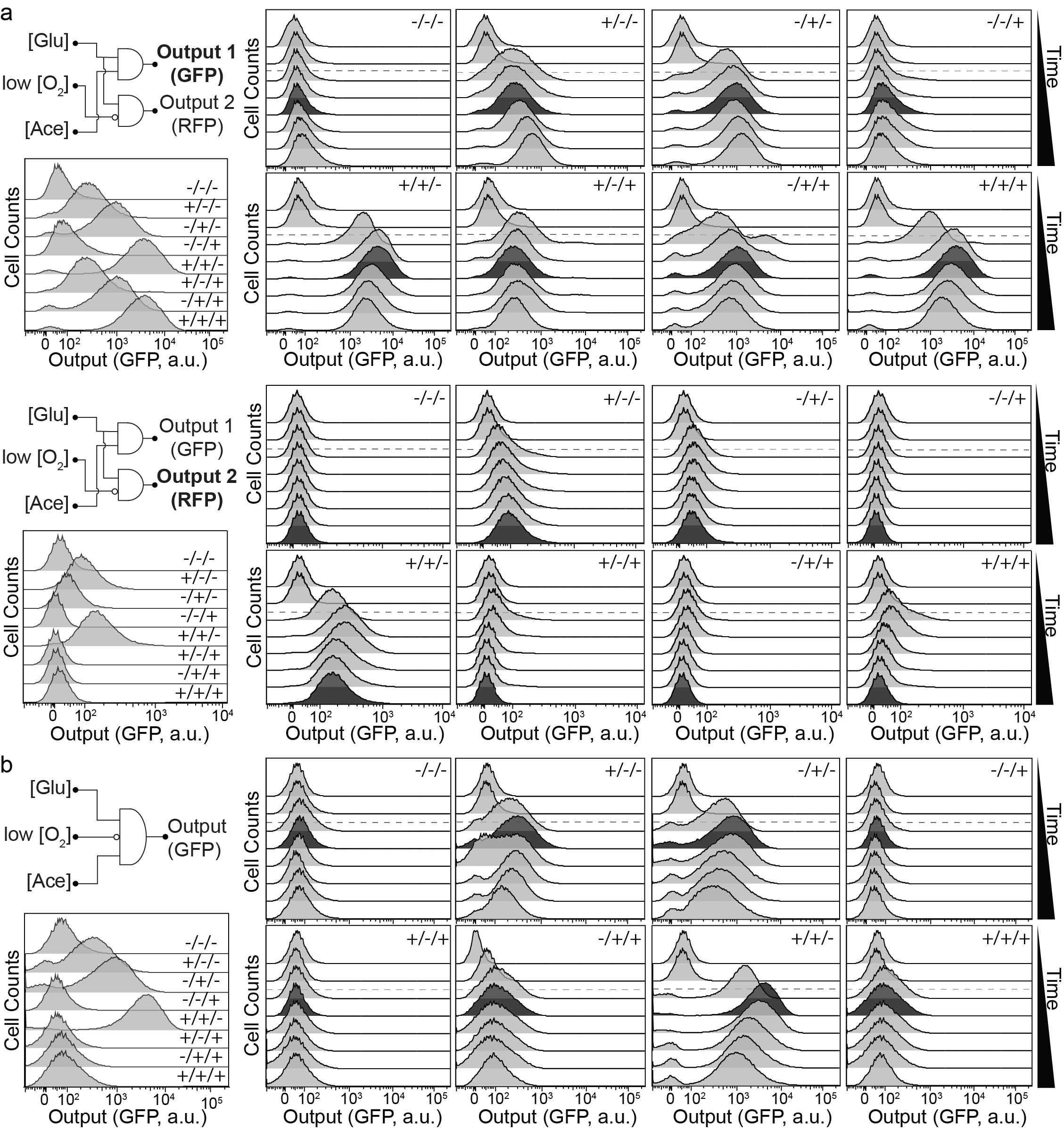
**

**Appendix Figure S10: Cytometry distributions corresponding to 3-input logic circuits.** Shown are the cytometry distributions for the two circuits shown in Figure 2. **(a)** GFP (top) and RFP (bottom) fluorescence of the 3-input, 2-output circuit. **(b)** GFP fluorescence of the 3-input, 1-output circuit. Below each circuit’s logic diagram are the cytometry distributions of the respective circuit in each condition, as shown in Figure 2g/j. To the right are shown the dynamic responses of the circuits in each condition at time intervals of 1 hour. Dashed lines indicate the time of induction with 30 mM acetate, 0.8% glucose, or anaerobic growth conditions. Darkened distributions in the dynamic responses correspond to the distributions shown at the left. Symbols (+/+/+) correspond to (Glucose/Acetate/Oxygen) sensors, respectively, and signify the active (+) or inactive (-) state each corresponding sensor promoter.


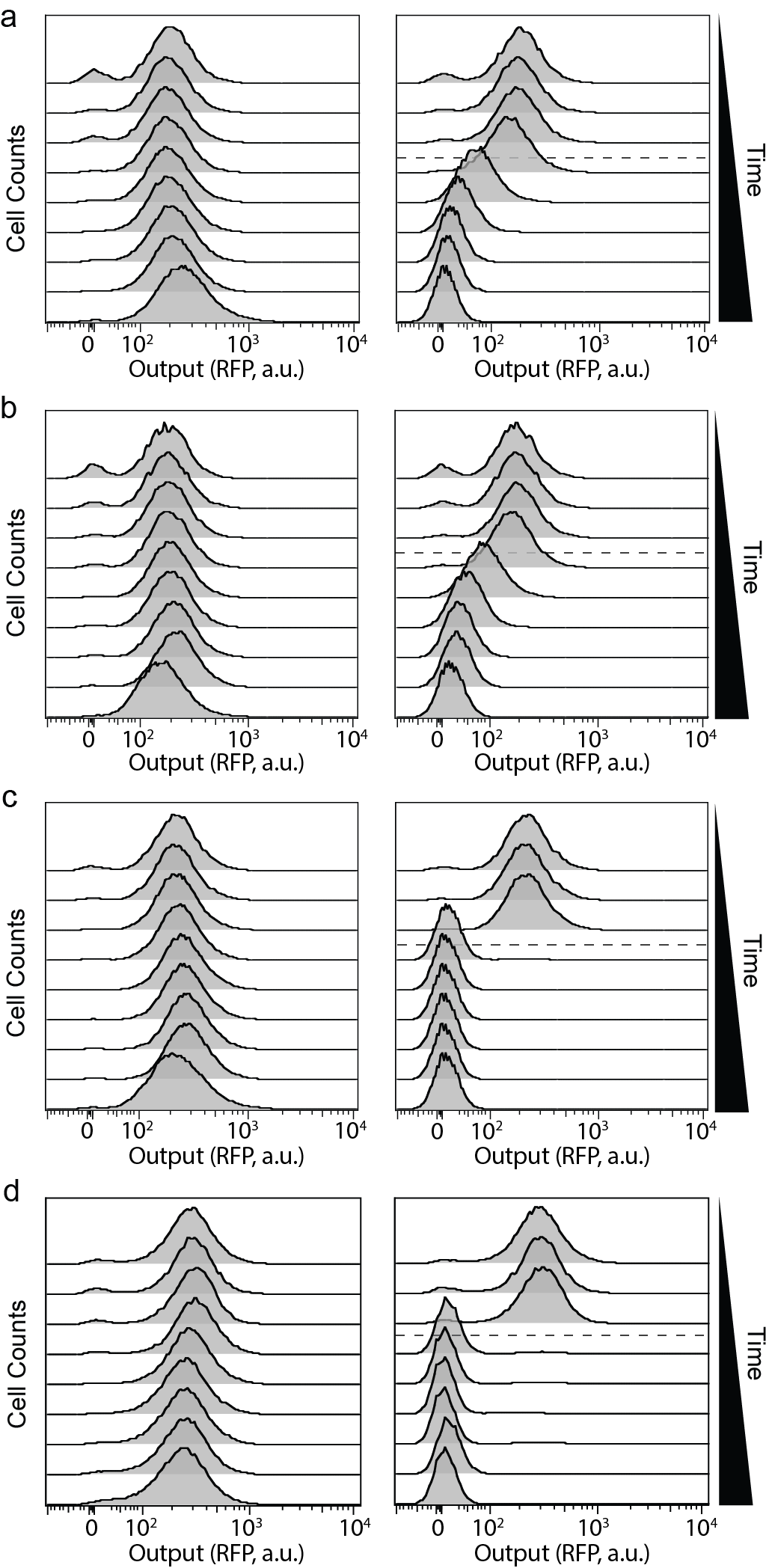


**Appendix Figure S11: Cytometry distributions corresponding to RFP repression by CRISPRi, sRNA, and *mf*-LON.** Shown are the cytometry distributions corresponding to the data in Figure 3c. Each culture contains a plasmid that constitutively expresses RFP (pFM1171) and a plasmid expressing either **(a)** dCas9 and sgRNA (pFM1136), **(b)** sRNA (pFM855), **(c)** protease *mf*-LON (pFM811), or **(d)** dCas9 and a transcriptional fusion of sgRNA and *mf*-LON (pFM1174) targeting RFP. Each of the left figures are uninduced cultures and each of the right figures are the induced cultures. The dashed line represents the time of induction. Time intervals are 1 hour. Cultures were grown in MM containing 0.8% glycerol and were induced with either 1 mM IPTG or 25 uM DAPG, as indicated. Maps for the plasmids listed here can be found in Appendix Figure S16.

**
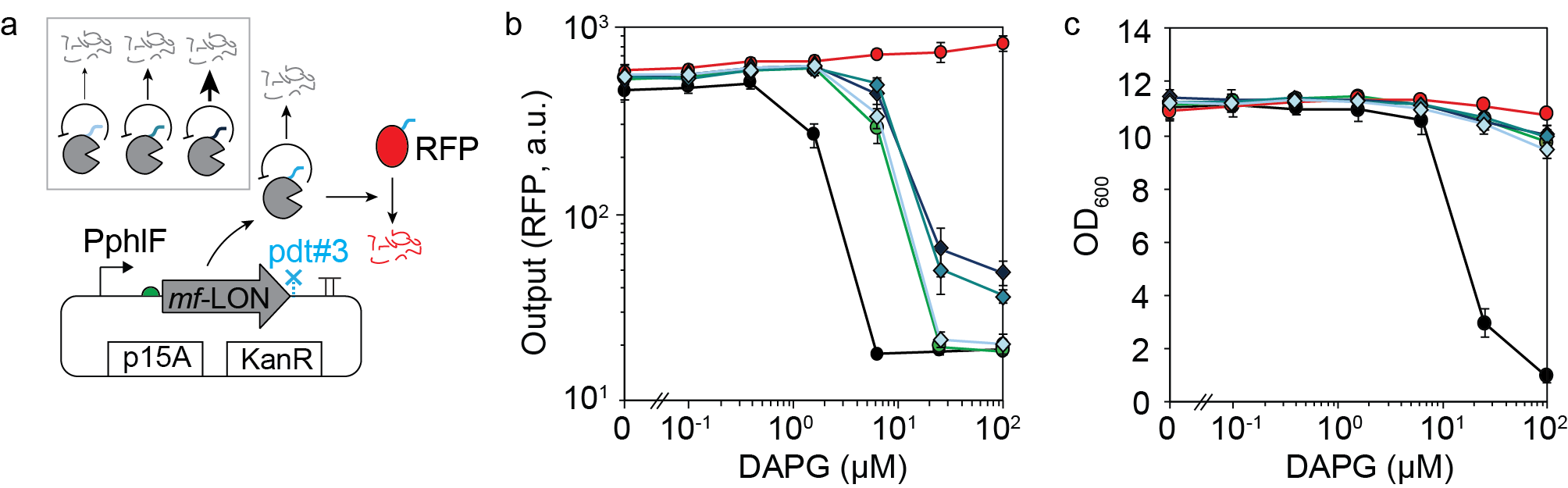
**

**Appendix Figure S12: Tuning *mf*-LON expression to reduce toxicity. (a)** Shown is a scheme in which *mf*-LON protease is expressed from a DAPG-inducible promoter (PphlF) on a p15A plasmid and degrades proteins fused to the pdt#3 degradation tag (blue), including itself and a constitutively-expressed red fluorescent protein (RFP). The inset shows how the strength of the pdt#3 tag on *mf*-LON changes the rate of auto-degradation ([Cameron & Collins, 2014](#_ENREF_3)). The graphs on the right show the change in RFP fluorescence **(b)** and final OD_600_ **(C)** of cell cultures constitutively expressing RFP and carrying plasmids expressing variants of *mf*-LON at different levels of induction. Black and green circles show constructs expressing *mf*-LON from a strong and weak RBS, respectively. Blue diamonds show constructs expressing *mf*-LON from the same weak RBS and variable strength pdt#3 tags. Darker shades of blue represent faster degradation rates for the tags. Red circles are cultures that contain no *mf*-LON. The lightest blue diamonds represent the culture carrying plasmid pFM811, which was used to test degradation of RFP and *pta* gene product in Figure 3. RFP fluorescence was assayed 6 hours after induction during growth on MM containing 0.8% glycerol in 96-well plates and OD_600_ was assayed after 20 hours of growth. Error bars represent one standard deviation of three experiments done on different days.


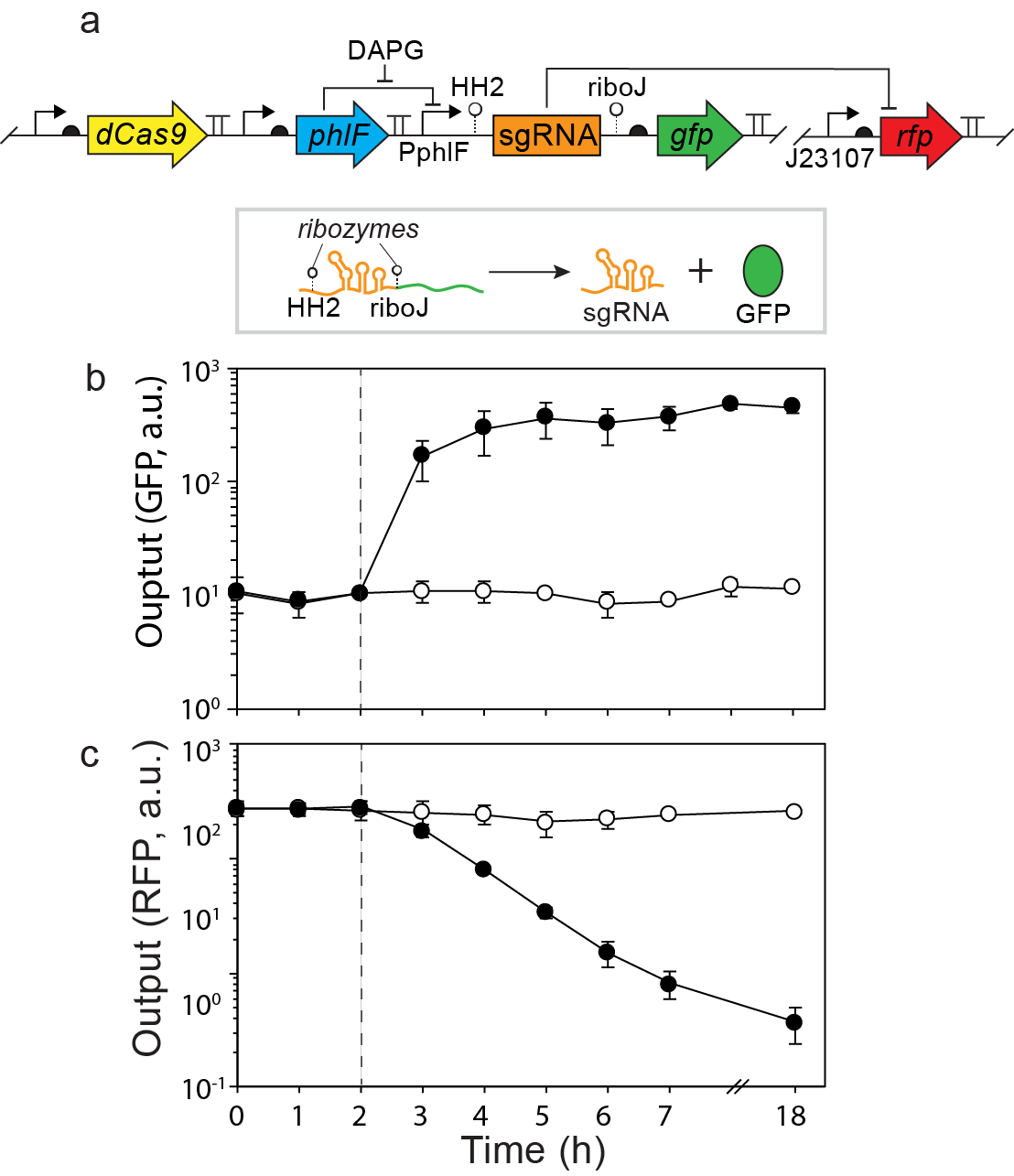


**Appendix Figure S13: Fusion of sgRNA and CDS transcripts generates functional sgRNA and protein. (a)** Shown is the genetic diagram for a combined sgRNA/*gfp* system (pFM1149) identical to the combined sgRNA/*mf*-LON system (pFM1174) shown in Figure 3c except that the *mf*-LON gene has been replaced with *gfp*. In both plasmids, an RFP-targeting sgRNA is transcriptionally fused to a CDS. Following transcription, the HH2 ribozyme ([Ghodasara & Voigt, 2017](#_ENREF_10)) generates a clean 5' end on the sgRNA and riboJ ([Lou et al, 2012](#_ENREF_16)) cleaves the CDS transcript from the 3' end of the sgRNA. RFP is constitutively expressed from plasmid pFM1171. Shown are the median **(b)** GFP and **(c)** RFP fluorescence of the pFM1149 strain before and after induction. The dotted line indicates when 25 µM DAPG is added to the media. Error bars are one standard deviation from three replicates measured on different days.


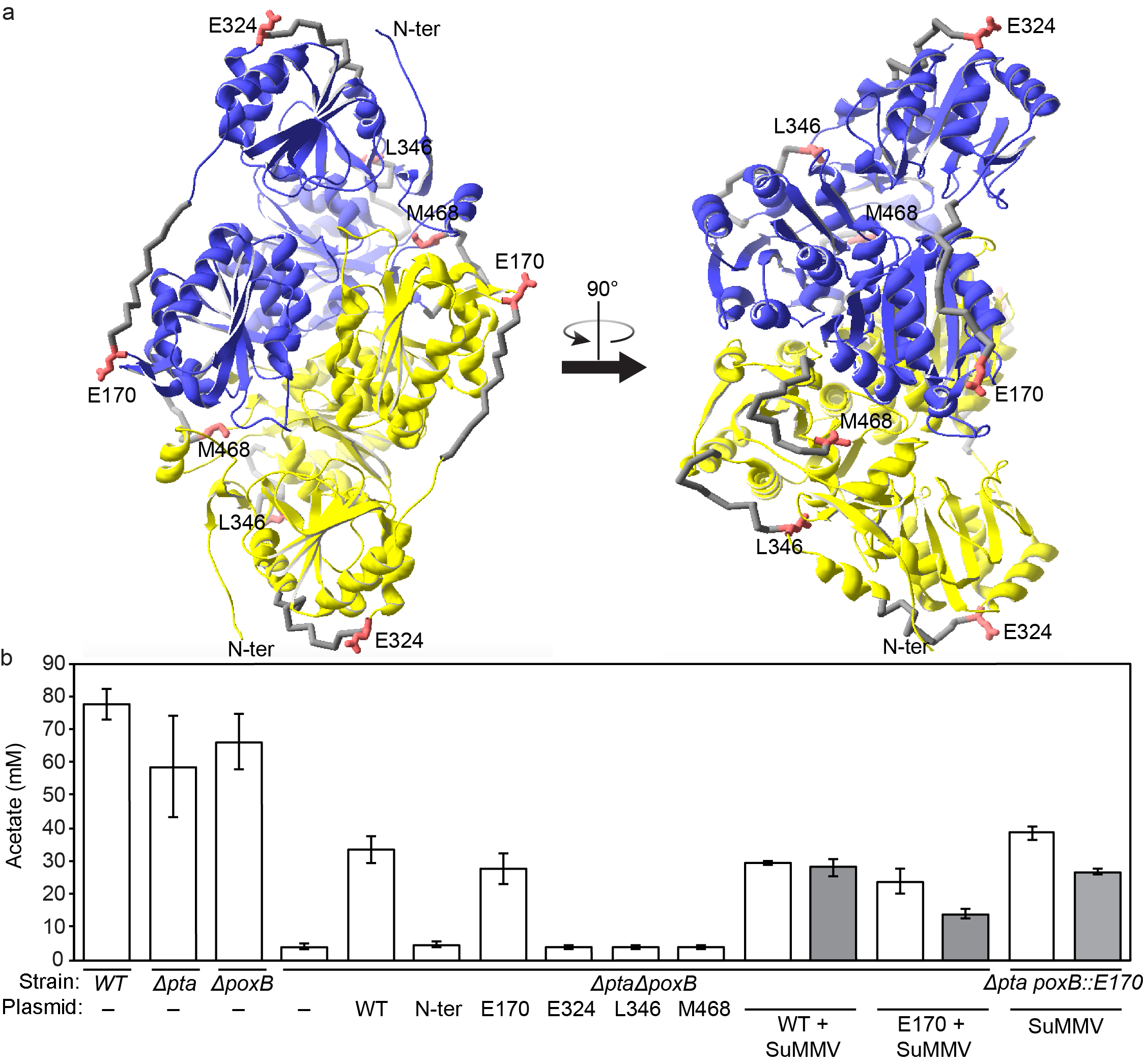
**Appendix Figure S14: SuMMV degradation tag insertion into PoxB structure. (a)** Structure of the pyruvate oxidase (PoxB) dimer (PDB# 3EY9)([Neumann et al, 2008](#_ENREF_18)) with the screened insertion sites for the SuMMV cleavage site and N-terminal degron (EEIHLQ-FLFVQ) highlighted in grey. The native first residue of each site is numbered and shown in red. PoxB monomers are shown in yellow and purple. **(b)** Shown is the acetate produced by *E. coli* cultures after 20 hours of growth in 3 ml of MM+1.6% glucose in culture tubes. Cultures include wild-type MG1655 (WT), knockout mutants of *pta* (*E. coli* MG1655 Δ*pta*) and *poxB* (*E. coli* MG1655 Δ*poxB)*, and double knockout strains (*E. coli* MG1655 Δ*pta*Δ*poxB*) containing either no plasmid (-) or BACs encoding variants of *poxB* under endogenous regulation. The *poxB* variants include native *poxB* (WT) and *poxB* containing the SuMMV degradation tag inserted at the N-terminus (N-ter) or various exposed locations on the protein (E170, E324, L346, M468). BACs encoding the native (WT; pFM919) variant of *poxB* and the E170 variant (pFM1047) were also co-transformed with a plasmid expressing SuMMV protease (pFM1069) from a DAPG-inducible PphlF promoter (SuMMV). The E170 mutation was finally inserted into a *E. coli* MG1655 Δ*pta* strain by recombineering, generating the strain *E. coli* MG1655 Δ*pta poxB::E170* and transformed with the SuMMV plasmid. These cultures were grown uninduced (white) and induced (shaded) with 25 µM DAPG. Error bars are one standard deviation of three experiments done on different days.


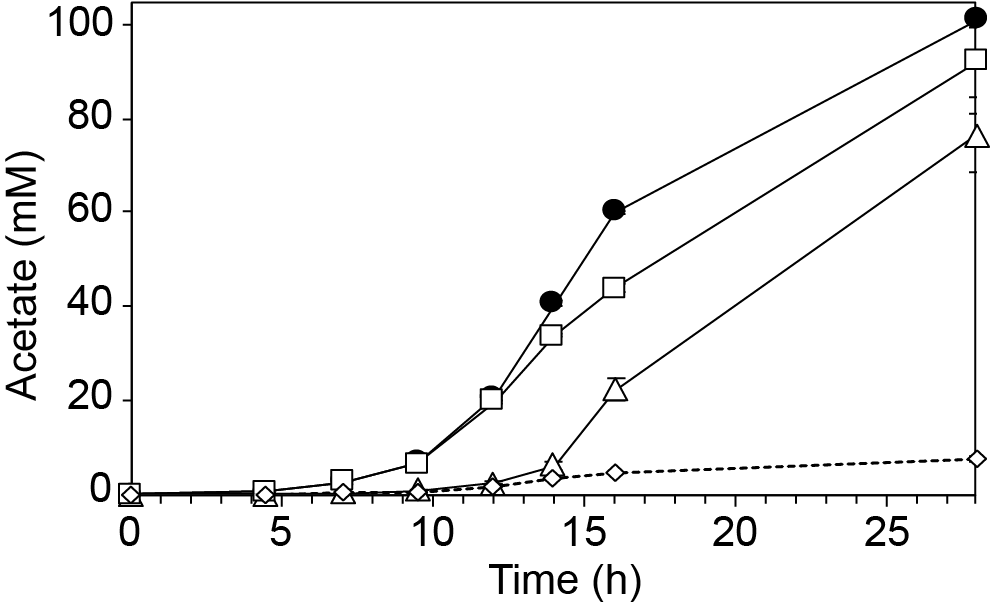


**Appendix Figure S15: Effect of *pta* and *poxB* deletions on the dynamics of acetate production. (a)** Shown is the dynamic accumulation of acetate by *Escherichia coli* MG1655 Δ*glnL* (filled circles), Δ*glnL*Δ*pta* (open triangles), Δ*glnL*Δ*poxB* (open squares), and Δ*glnL*Δ*pta*Δ*poxB* (open diamonds) grown on MM containing 1.6% glucose. Error bars are one standard deviation of three experiments done on different days.


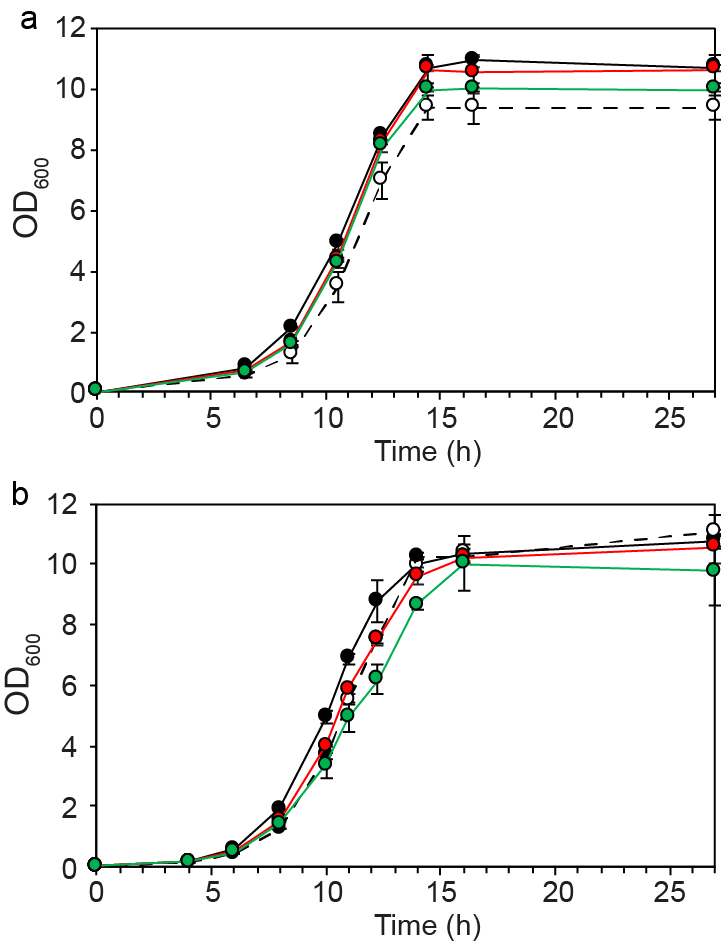


**Appendix Figure S16: Growth curves of circuit strains. (a)** Shown are the measured cell culture optical densities (OD_600_) corresponding to Figure 4c. Solid black circles and line represents *E. coli* MG1655*∆glnL∆pta poxB::E170* containing no plasmids. Solid green circles and lines represent MG1655*∆glnL∆pta poxB::E170* containing plasmids pFM1330 and pFM1331. Solid red circles and lines represent MG1655*∆glnL∆pta* containing plasmids pFM1329 and pFM1331. Empty circles connected by the dashed line represent MG1655*∆glnL∆pta∆poxB.* **(b)** Shown are the measured cell culture optical densities (OD_600_) corresponding to Figure 4f. Solid black circles and line represents *E. coli* MG1655*∆glnL∆poxB pta::pdt3* containing no plasmids. Solid green circles and lines represent *E. coli* MG1655*∆glnL∆poxB pta::pdt3* containing plasmids pFM1333 and pFM1335. Solid red circles and lines represent *E. coli* MG1655*∆glnL∆poxB* containing plasmids pFM1333 and pFM1334. Empty circles connected by the dashed line represent MG1655*∆glnL∆pta∆poxB.* Colors of the lines and dots correspond with those in Figure 4. Error bars represent one standard deviation from three different experiments.


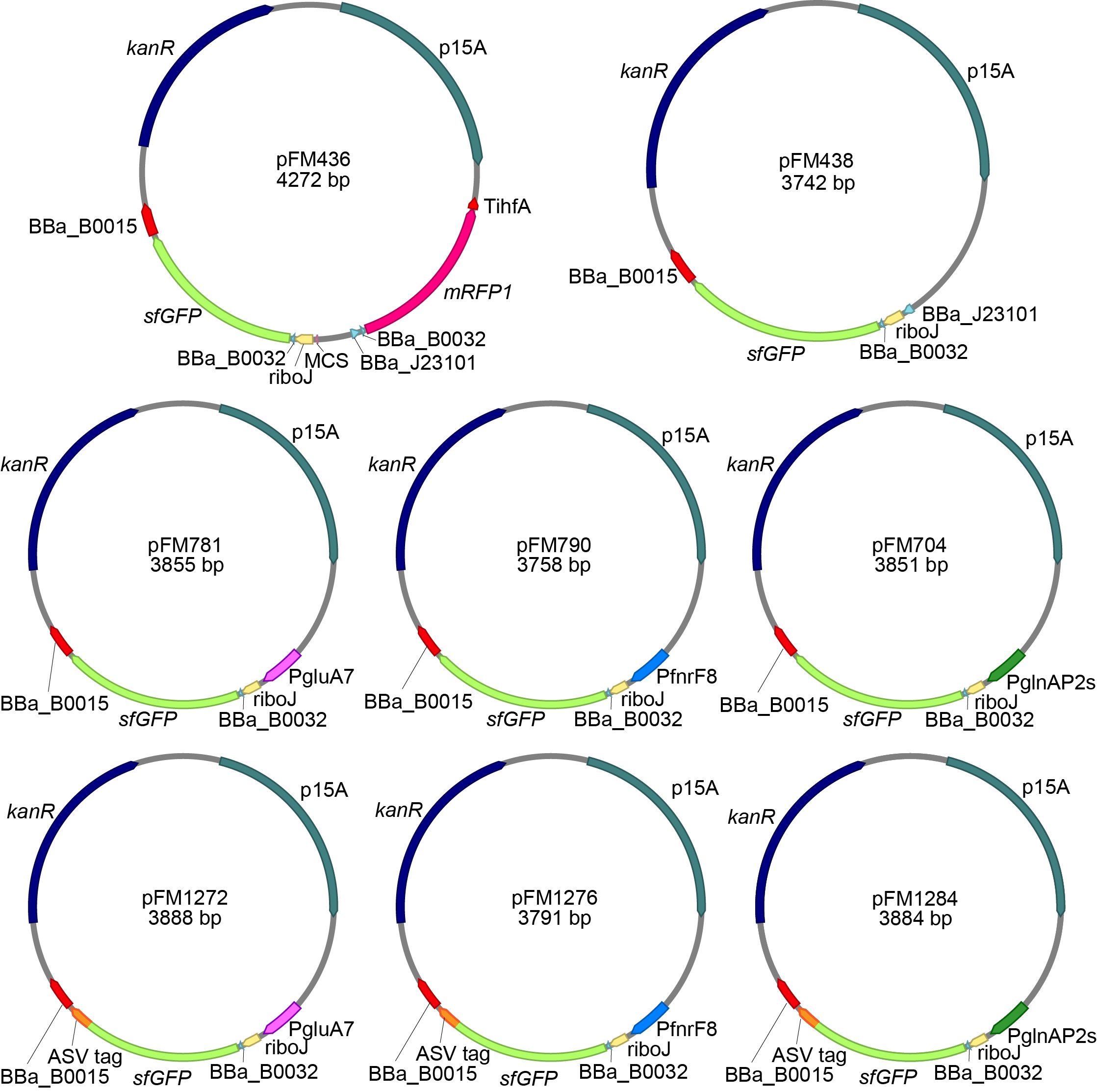


**Appendix Figure S17:** **Plasmid maps.** Shown are the plasmids used to generate data in Figure 1 and Figure 2a,b,c. pFM436 is the screening plasmid for sensor libraries and contains a multiple cloning site (MCS) for directional promoter insertion (Methods). pFM438 is the standard measurement plasmid. pFM781, pFM790, and pFM704 are the sensor plasmids for the PgluA7, PfnrF8, and PglnAP2s promoters, respectively, used to generate sensor response data in Figure 1. pFM1272, pFM1276, and pFM1284 are the sensor plasmids for PgluA7, PfnrF8, and PglnAP2s, respectively, with an ASV degradation tag on the C-terminus of *sfGFP* for Figure 2a,b,c. The *sfGFP* gene encodes a superfolder GFP protein (Appendix Table S2). Annotated sequence files are available in the authors' Github repository (https://github.com/VoigtLab/promoter-library-design-tool).

**
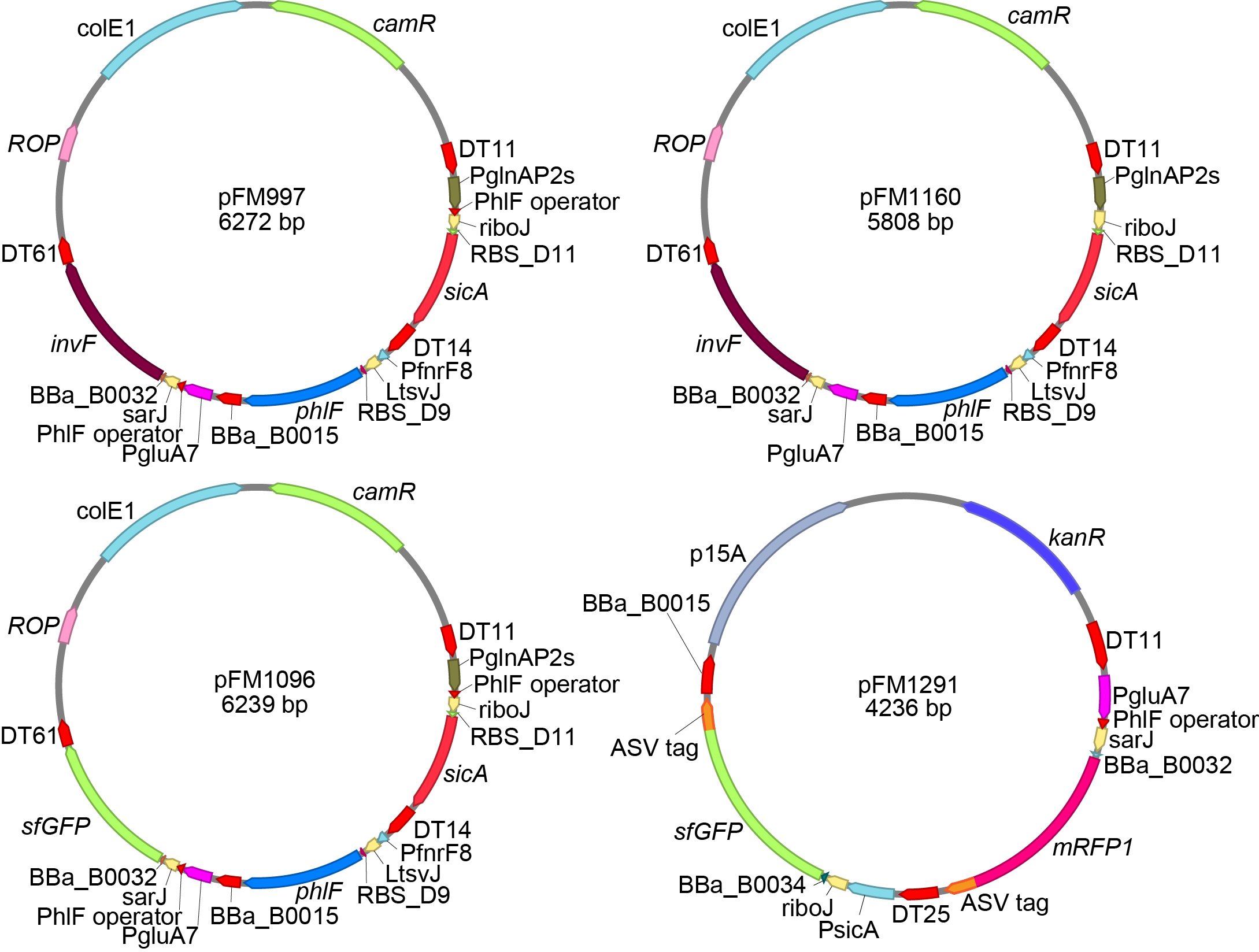
**

**Appendix Figure S17 (continued):** **Plasmid maps.** Shown are the plasmids used to generate data in Figure 2e/g/h/j. pFM1160 and pFM1291 were used to generate the GFP and RFP output data in Figure 2e/g. pFM997 and pFM1291 were used to generate the 3-input AND gate data in Figure 2h/j. pFM1096 and pFM1160 were used to generate data for Appendix Figure S9.


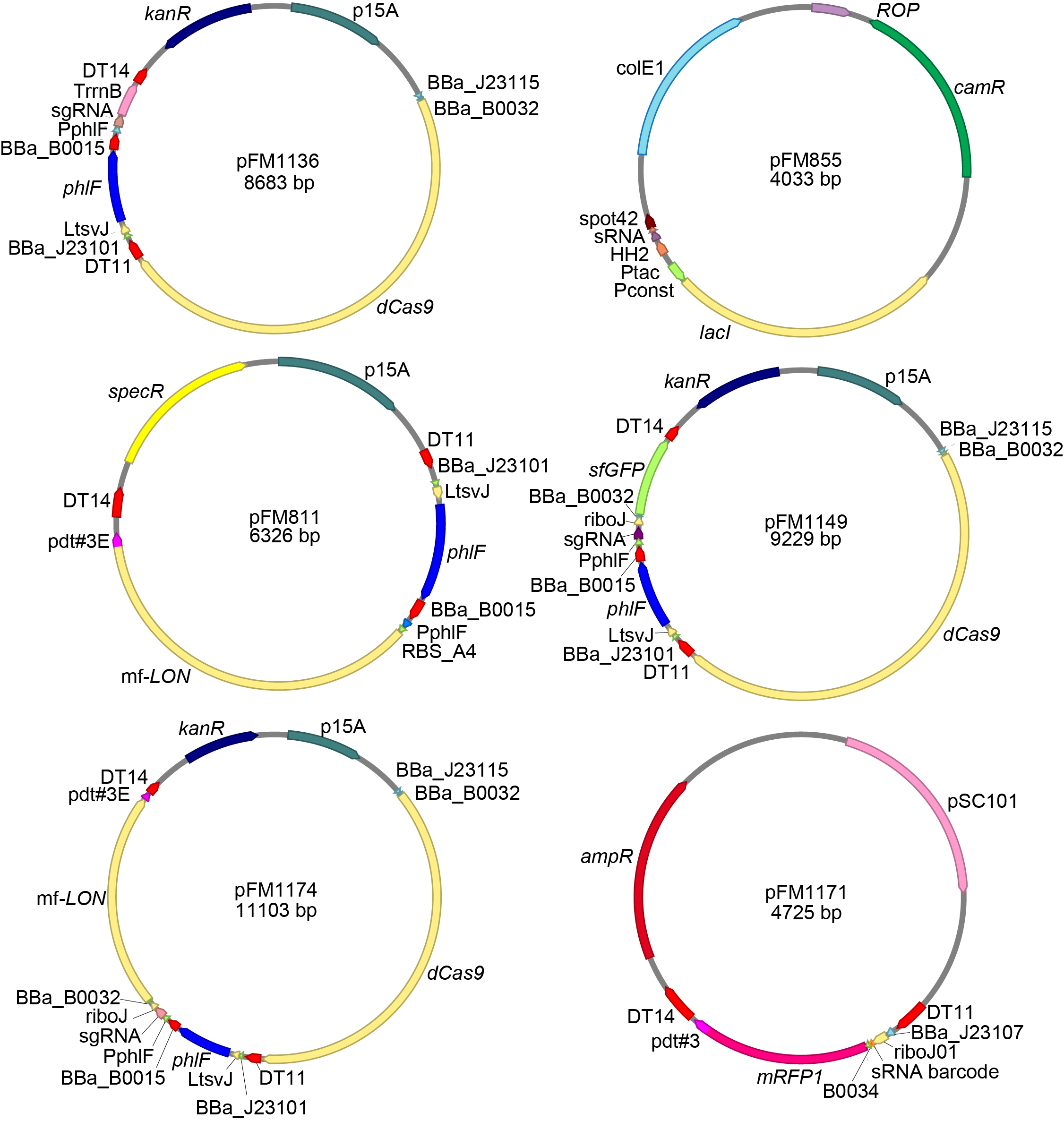


**Appendix Figure S17 (continued):** **Plasmid maps.** Shown are the plasmids used to generate data in Figure 3b,c. pFM1136 is the CRISPRi output plasmid, pFM855 is the sRNA output plasmid, pFM811 is the *mf*-LON output plasmid, and pFM1174 is the combination sgRNA+*mf*-LON output plasmid. pFM 1149 was used to generate data for Appendix Figure S13. pFM1171 was used to generate mRFP1 target gene, which is tagged with an *mf*-LON pdt#3E degradation tag and has an sRNA barcode sequence upstream of its RBS.

**
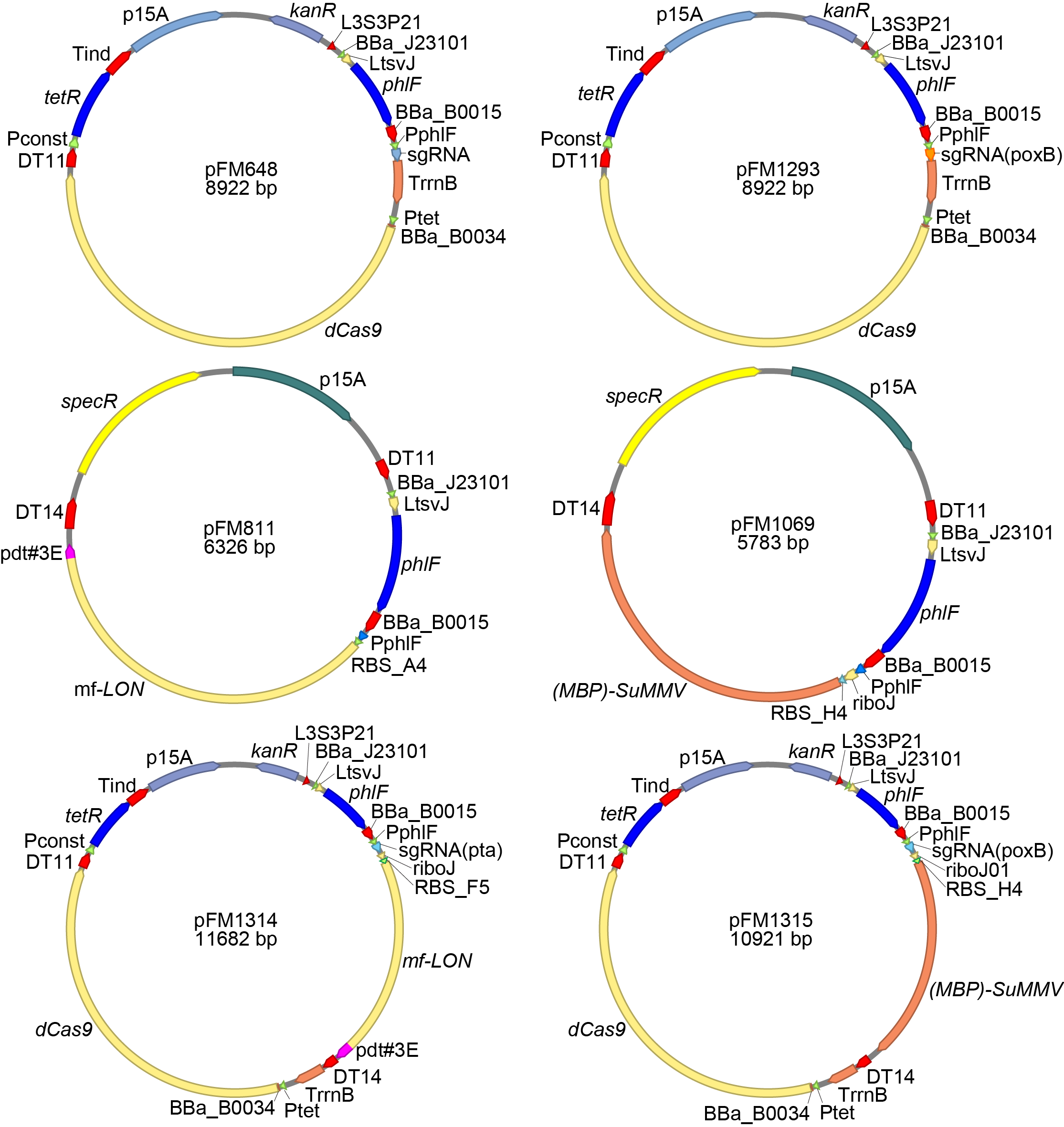
**

**Appendix Figure S17 (continued):** **Plasmid maps.** Shown are the plasmids used to generate data in Figure 3d. pFM648 is the sgRNA (CRISPRi) plasmid targeting *pta*. pFM1293 is the sgRNA (CRISPRi) plasmid targeting *poxB*. pFM811 is the *mf*-LON output plasmid. pFM1069 is the Maltose Binding Protein (MBP)-SuMMV fusion output plasmid. pFM1314 is the combination sgRNA(*pta*)+*mf*-LON output plasmid. pFM1315 is the sgRNA(*poxB*)+SuMMV output plasmid.


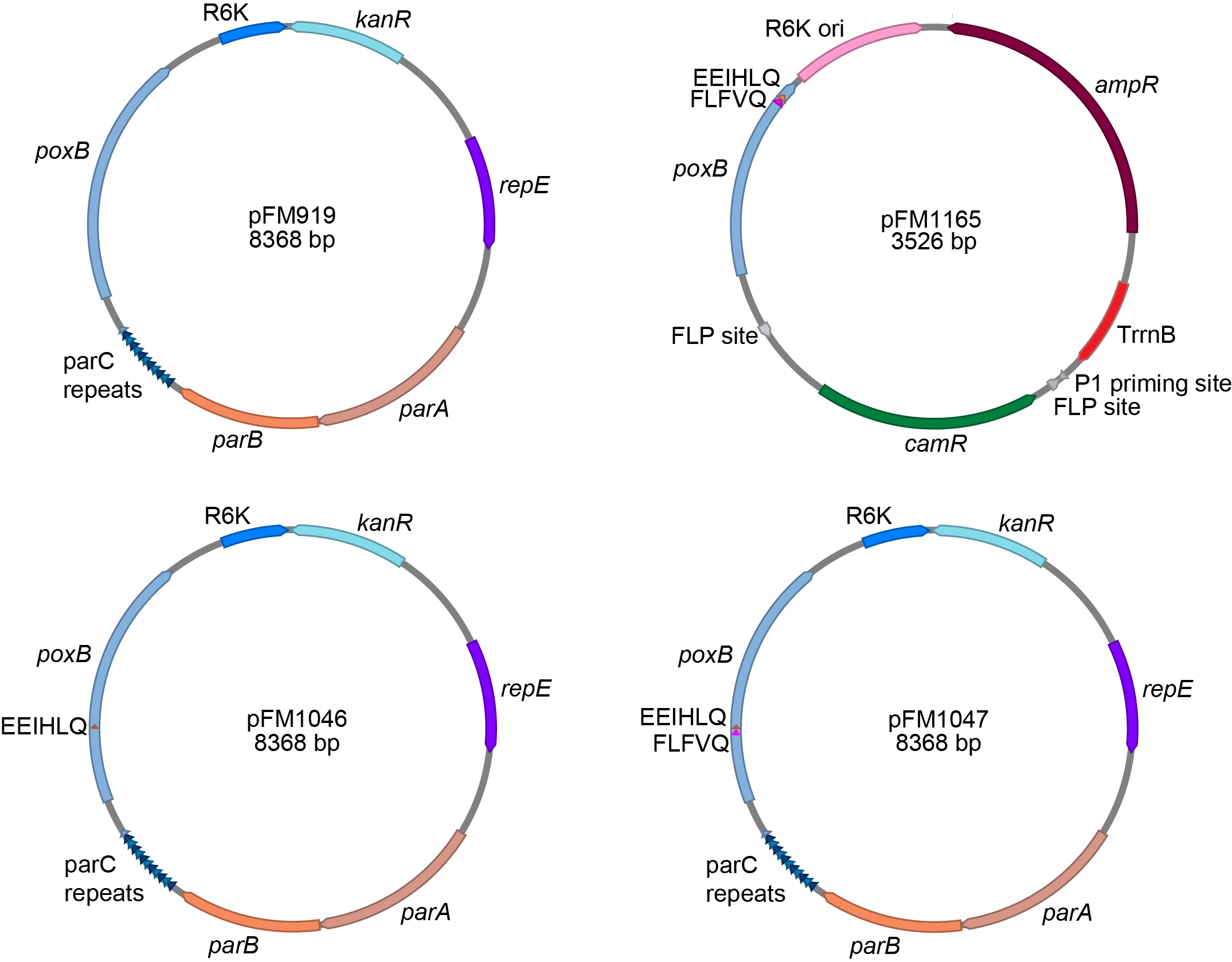


**Appendix Figure S17 (continued):** **Plamid maps.** Shown are the plasmids used to test the SuMMV tag (EEIHLQ-FLFVQ) in *poxB*. pFM1165 was used to generate the modified *poxB* sequence on the genome. pFM919 is the BAC containing the Wild-Type *poxB* sequence expressed from native regulation. pFM1046 is the modified *poxB* sequence containing only the SuMMV cleavage site (EEIHLQ). pFM1047 is the modified *poxB* sequence (*poxB*::E170) containing both the SuMMV cleavage site (EEIHLQ) and the N-terminal degron (FLFVQ) expressed from native regulation.


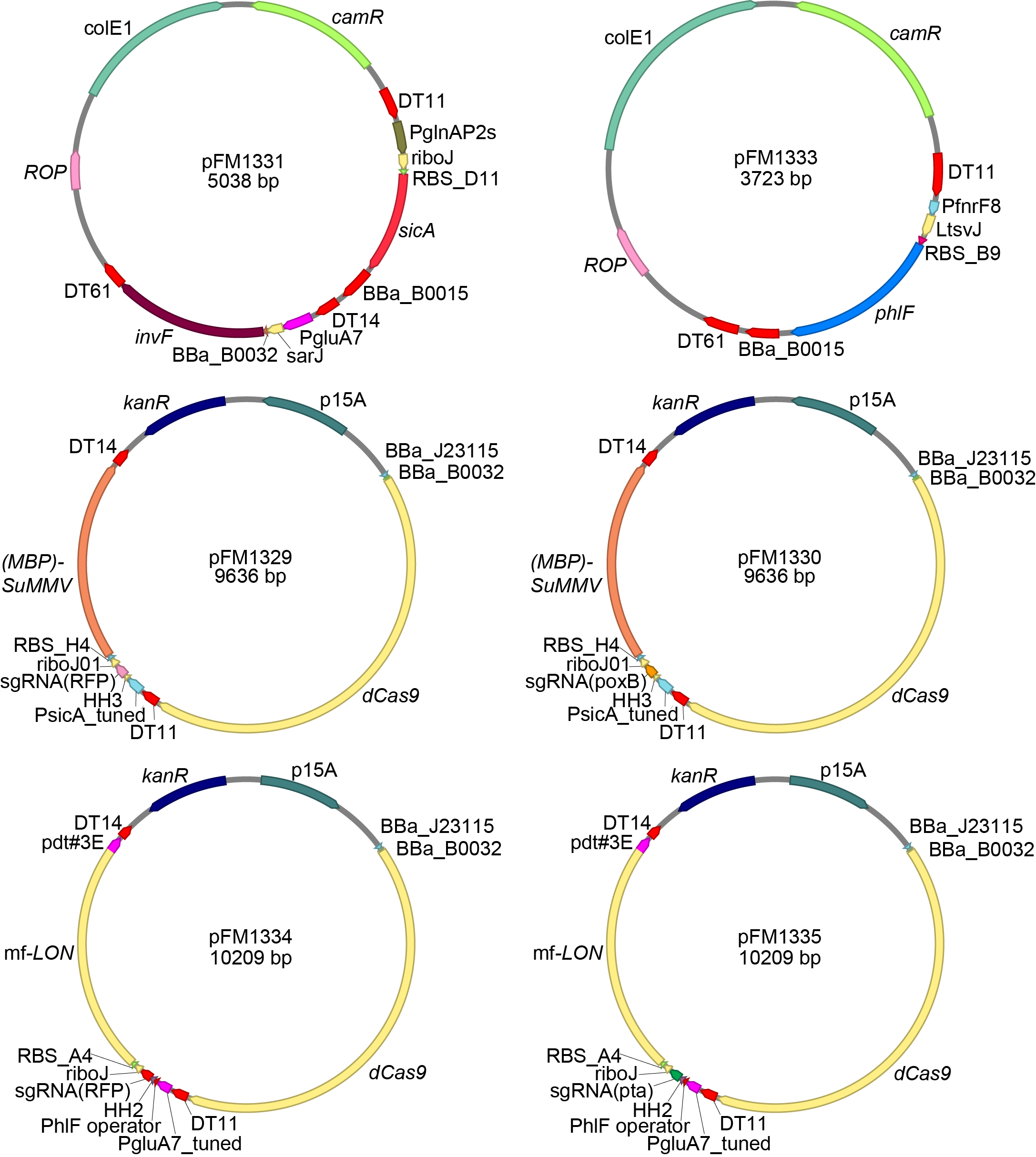


**Appendix Figure S17 (continued):** **Plamid maps.** Shown are the plasmids used to generate data in Figure 4c and 4f. pFM1331 and pFM1329 are used to generate the RFP-targeting output for Figure 4c. pFM1331 and pFM1330 are used to generate the *poxB*-targeting output for Figure 4c. pFM1333 and pFM1334 are used to generate RFP-targeting output for Figure 4f. pFM1333 and pFM1335 are used to generate the *pta*-targeting output for Figure 4f.

**Appendix Table S1: Growth rates of acetate pathway mutants. ^†^**

| Strain | Specific Growth Rate (hr^-1^)^‡^ | | Final OD_600_^‡∗^ |
| --- | --- | --- | --- |
| MG1655  MG1655Δ*glnL* | | 0.59 ± 0.01  0.59 ± 0.02 | 10.29 ± 0.47  10.91 ± 0.96 |
| MG1655Δ*glnL*Δ*pta* | | 0.50 ± 0.01 | 10.48 ± 0.83 |
| MG1655Δ*glnL*Δ*ackA* | | 0.34 ± 0.05 | 1.63 ± 0.48 |
| MG1655Δ*glnL*Δ*poxB* | | 0.58 ± 0.02 | 10.85 ± 1.19 |
| MG1655Δ*glnL*Δ*ptaΔackA* | | 0.49 ± 0.02 | 9.91 ± 1.22 |
| MG1655Δ*glnL*Δ*pta*Δ*poxB* | | 0.50 ± 0.01 | 10.69 ± 1.00 |
| MG1655Δ*glnL*Δ*pta*Δ*ackA* Δ*poxB::KanR* | | 0.51 ± 0.01 | 10.54 ± 1.76 |
| MG1655Δ*glnL*Δ*poxB pta::pdt3* | | 0.59 ± 0.01 | 10.53 ± 0.50 |
| MG1655Δ*glnL*Δ*pta poxB::E170* | | 0.53 ± 0.05 | 10.91 ± 0.21 |
| MG1655Δ*glnL pta::pdt3 poxB::E170* | | 0.58 ± 0.01 | 10.35 ± 0.46 |
| †Measured in 30 ml shake flask cultures containing minimal media with 1.6% glucose, grown at 37˚C at 250 RPM with a 1 inch throw.  ‡Error is one standard deviation of three independent experiments.  ^∗^After 27 hours of growth. | | |  |
|  | | |  |

**Appendix Table S2:** Genetic parts used in this work.

| **Part** | **Type** | **Sequence** | **Ref.** |
| --- | --- | --- | --- |
| PgluA7 | Promoter | TTGACAGCTAGCTCAGTCCTAGGTATAAATGTGATCTAGATCACATTT | This work |
| PgluA7* | Promoter | TTGACAGCTAGCTCAGTCCTAGGTATAATATCAGGTCCACATCTGGTC | This work |
| PgluA7_PhlFoperator | Promoter | TTGACAGCTAGCTCAGTCCTAGGTATAAATGTGATCTAGATCACATTT ATGATACGAAACGTACCGTATCGTTAAGGT | This work |
| PfnrF8 | Promoter | TTTGATTTACATCAATTACGGCTAGCTCAGTCCTAGGTATTATGCTAGCTA | This work |
| PfnrF8* | Promoter | TAATAAGAGGTGGGATTACGGCTAGCTCAGTCCTAGGTATTATGCTAGCTA | This work |
| PglnAP2 | Promoter | CAAAGGTCATTGCACCAACATGGTGCTTAATGTTTCCATTGAAGCACTATATTGGTGCAACATTCACATCGTGGTGCAGCCCTTTTGCACGATGGTGCGCATGATAACGCCTTTTAGGGGCAATTTAAAAGTTGGCACAGATTTCGCTTTATCTTTTTTACGGCGACACGGCCAAAATAATTGCAGATTTCGTTACCACGACGACCATGACCAATCCAGGAGAGTTAAAGTATGTCCGCTGAACACGTACTG | ([Farmer & Liao, 2001](#_ENREF_7)) |
| PglnAP2s | Promoter | CAAAGGTCATTGCACCAACATGGTGCTTAATGTTTCCATTGAAGCACTATATTGGTGCAACATTCACATCGTGGTGCAGCCCTTTTGCACGATGGTGCGCATGATAACGCCTTTTAGGGGCAATTTAAAAGTTGGCACAGATTTCGCTTTATCTTTTTTA | This work |
| BBa_J23101 | Promoter | TTTACAGCTAGCTCAGTCCTAGGTATTATGCTAGC | Registry of Biological Parts |
| BBa_J23115 | Promoter | TTTATAGCTAGCTCAGCCCTTGGTACAATGCTAGC | Registry of Biological Parts |
| PphlF | Promoter | TCTGATTCGTTACCAATTGACATGATACGAAACGTACCGTATCGTTAAGGT | ([Stanton et al, 2014b](#_ENREF_28)) |
| Ptac | Promoter | TGTTGACAATTAATCATCGGCTCGTATAATGTGTGGAATTGTGAGCGCTCACAATT | ([Nielsen et al, 2016](#_ENREF_19)) |
| Ptet | Promoter | TTTTTTCCCTATCAGTGATAGAGATTGACATCCCTATCAGTGATAGAGATAATGAGCAC | ([Nielsen & Voigt, 2014](#_ENREF_20)) |
| PyeiW | Promoter | CGAAAAATTCCTGTAGTCGAAAATGTCAAAAATATCGCGACAAAGTACTCGCGACGGGCCTTTCGGGCAAGTGGTATTCGCACTTTTGCTGGTGCAAAAAAGGTGGTACTGTGCGCGCTCATCAATCCGGTGGTTAACCTTAAGAGAACAACG | ([Raghavan et al, 2011](#_ENREF_23)) |
| PcyA | Promoter | GCAAACCGTAAAATGAGGTCTGGCAGTGGATCCTGACAGGCGTTTCACGCCGTTGTAATAAGGAATTTACAGAGAATAAACGGTGCTACACTTGTATGTAGCGCATCTTTCTTTACGGTCAATCAGCAAGGTGTTAAATTGATCACGTTTTAGACCATTTTTTCGTCGTGAAACTAAAAAAACCAGGCGCGAAAAGTGGTAACGGTTACCTTTGACATACGAAATATCCCGAATGCCGCGTGTTACCGTTGATGTTGGCGGAATCACAGTCATGACGGGTAGCAAATCAGGCGATACGT | ([Aiba, 1985](#_ENREF_2)) |
| ParaJ | Promoter | CAAACTGGAAAGTACGTTTGCAGTGAAATAACTATTCAGCAGGATAATGAATACAGAGGGGCGAATTATCTCTTGGCCTTGCTGGTCGTTATCCTGCAAGCTATCACTTTATTGGCTACGGTGATTGGTAGCCGTTCTGGTGGTTGTGATGGTGGT | ([Hendrickson et al, 1990](#_ENREF_11)) |
| Pgalp2 | Promoter | GTCGGTAGTGCTGACCTTGCCGGAGGCGGCCTTAGCACCCTCTCCGGCCAACGGTTCGACGCATGCAGGCATGAAACCGCGTCTTTTTTCAGATAAAAAGCGCAATCATTCATAAACCCTCTGTTTTATAATCACTTAATCGCGCATAAAAAACGGCTAAATTCTTGTGTAAACGATTCCACTAATTTATTCCATGTCACACTTTTCGCATCTTTGTTATGCTATGGTTATTTCATACCATAAGCCTAATGGAGCGAATT | ([Lewis & Adhya, 2015](#_ENREF_14)) |
| PompR | Promoter | CTCGTTGATTCCCTTTGTCTGTTTGATAATGCGCACATTGGGTATAACGTGATCATATCAACAGAATCAATAATGTTTCGCCGAATAAATTGTATACTTAAGCTGCTGTTTAATATGCTTTGTAACAATTTAGGCTGAAATTCATACCAGATTTAGCTGGTGACGAACGTGAGCTTTTTTAAGAATACACGCTTACAAATTGTTGCGAACCTTTGGGAGTACAAACA | ([Huang et al, 1992](#_ENREF_12)) |
| Pspf | Promoter | GATTCGCCTGAACATGCCTTTTTTCGTAAGTAAGCAACATAAGCTGTCACGTTTTGTGATGGCTATTAGAAATTCCTATGCAACAACTGAAAAAAAATTACAAAAAGTGCTTTCTGAACTGAACAAAAAAGAGTAAAGTTAGTCGC | ([Collado-Vides et al, 1991](#_ENREF_5)) |
| PtrxA | Promoter | TGCAGGGCGAAGTCGGAAAACTTCTGTTCTGTTAAATGTGTTTTGCTCATAGTGTGGTAGAATATCAGCTTACTATTGCTTTACGAAAGCGTATCCGGTGAAATAAAGTCAACCTTTAGTTGGTTAATGTTACACCAACAACGAAACCAACACGCCAGGCTTATTCCTGTGGAGTTATAT | ([Sa et al, 1997](#_ENREF_24)) |
| PnirB | Promoter | TTGCTCATGCCGGACGGCACTATCGTCGTCCGGCCTTTTCCTCTCTTCCCCCGCTACGTGCATCTATTTCTATAAACCCGCTCATTTTGTCTATTTTTTGCACAAACATGAAATATCAGACAATTCCGTGACTTAAGAAAATTTATACAAATCAGCAATATACCCATTAAGGAGTATATAAAGGTGAATTTGATTTACATCAATAAGCGGGGTTGCTGAATCGTTAAGGTAGGCGGTAATAGAAAAGAAATCGAGGCAAAA | ([Oxer et al, 1991](#_ENREF_21)) |
| PnarG | Promoter | ACCGTTACTCGTCATACTTCGGGTTACATGTGCTGCGGCTGCGTTCATTCACCCCAGTCACTTACTTTAGTAAGCTCCTGGGATTCATTCACTTGCCGCCTTCCTGTAAACCGAATTATATAGAGTAAAATATTTGATTATCCTTTGCGCGGCATGATGTCGCGCTTTTTTTATGCGTCATTTAGTTACAACATACTAATGTTATATGGTTTATTTCGCCGGATTTCATTAAGAGCCATTAATATGTTACCCATGGGGAATACTCCTTAATACCCATCTGCATAAAAATCTTAATAGTTTAAATAACTACAGGTATAAAACGTCTTAATTTACAGTCTGTTATGTGGTGGCTGTTAATTATCCTAAAGGGGTATCTTAGGAATTTACTTTATTTTTCATCCCCATCACTCTTGATCGTTATCAATTCCCACGCTGTTTCAGAGCGTTACCTTGCCCTTAAACATTAGCAATGTCGATTTATCAGAGGGCCGACAGGCTCCCACAGGAGAAAACCG | ([Garcia et al, 2009](#_ENREF_9)) |
| PfdhF | Promoter | AATGTCTACCGCGTGATGGCTGTCACGCGGTATTTCGTTTCGTCACGTCAAAACTGACGACAGCCTGTTTTTCGTCAGAGTTTTGAATAAATAGTGCCCGTAATATCAGGGAATGACCCCAAATAAAATGTGGCATAAAAGATGCATACTGTAGTCGAGAGCGCGTATGCGTGATTTGATTAACT | ([Salmon et al, 2003](#_ENREF_25)) |
| PyjiH | Promoter | CTCGTTTTCGTCCCTGGATTTTCTGTTTTTATTTACTGATGTCATTGTCACTTGGTTTATGAATTTTCTGCAACTCAATCCTGCGCAGAGCCTTTTCCACACAGCGTAATTTTTCCGCTATGCAAAAAACGCATAGCCAGAAACATACTTGCATTTTCCCCATACCCACATGGCTGATAAGGTTTTTCAGTTCCAGGAATTTTCCTGGCTCAAGCAACACACAAACACAAAAAAACA | ([Salmon et al, 2003](#_ENREF_25)) |
| PydfZ | Promoter | TTCATTTCCTCTCATCCCATCCGGGGTGAGAGTCTTTTCCCCCGCCTTATGGCTCATGCATGCATCAAAAAAGATGTGAGCTTGATCAAAAACAAAAAATATTTCACTCGACAGGAGTATTTATATTGCGCCCGTTACGTGGGCTTCGACTGTAAATCAGAAAGGAGAAAACACCT | ([Kang et al, 2005](#_ENREF_13)) |
| PdmsA | Promoter | TACCCAATTTTTCTGAATCTAAAAAGCGCCTGCGGGCGCTTTTTTTGTCTCCCTTTGATACCGAACAATAATTACTCCTCACTTACACGTAATACTACTTTCGAGTGAAAATCTACCTATCTCTTTGATTTTCAAATTATTCGATGTATACAAGCCTATATAGCGAACTGCTATAGAAATAATTACACAATACGGTTTGTTACTGGAATCAATCGTGAGCAAGCTTGAGTGAGCCATT | ([Kang et al, 2005](#_ENREF_13)) |
| PglpA | Promoter | TGATAGCCTCCGTGGCCCGTGGTCTTATTTATGATTAACAGCCTGATTCAGTGAGAGAACCTGCCGTTTCTTGAGTTGCCGCGATGTTAAGAAAACATTCATAAATTAAATGTGAATTGCCGCACACATTATTAAATAAGATTTACAAAATGTTCAAAATGACGCATGAAATCACGTTTCACTTTCGAATTATGAGCGAATATGCGCGAAATCAAACAATTCATGTTTTTACTATGGCTAAATGGTAAAAAACGAACTTCAGAGGGATAACA | ([Kang et al, 2005](#_ENREF_13)) |
| PsicA | Promoter | CCACAAGAAACGAGGTACGGCATTGAGCCGCGTAAGGCAGTAGCGATGTATTCATTGGGCGTTTTTTGAATGTTCACTAACCACCGTCGGGGTTTAATAACTGCATCAGATAAACGCAGTCGTTAAGTTCTACAAAGTCGGTGACAGATAACAGGAGTAAGTA | ([Moon et al, 2012](#_ENREF_17)) |
| PgluA7_tuned | Promoter | TTGAAAGCTAGCTCAGTCCTAGGGACAAATGTGATCTAGATCACATTT | This work |
| PsicA_tuned | Promoter | CCACAAGAAACGAGGTACGGCATTGAGCCGCGTAAGGCAGTAGCGATGTATTCATTGGGCGTTTTTTGAATGTTCACTAACCACCGTCGGGGTTTACTAACTGCATCAGATAAACGCAGTCGTTAAGTTCTACAAAGTCGGTGACAGATAACAGGAGTAAGTA | This work |
| Pconst | Promoter | GCGGCGCGCCATCGAATGGCGCAAAACCTTTCGCGGTATGGCATGATAGCGCCCGGAAGAGAGTCAATTCAGGGTGGTGAAT | This work |
| PhlF operator | Operator | ATGATACGAAACGTACCGTATCGTTAAGGT | ([Stanton et al, 2014a](#_ENREF_27)) |
| riboJ | Insulator | AGCTGTCACCGGATGTGCTTTCCGGTCTGATGAGTCCGTGAGGACGAAACAGCCTCTACAAATAATTTTGTTTAA | ([Lou et al, 2012](#_ENREF_16)) |
| ltsvJ | Insulator | AGTACGTCTGAGCGTGATACCCGCTCACTGAAGATGGCCCGGTAGGGCCGAAACGTACCTCTACAAATAATTTTGTTTAA | ([Liu & Zhang, 2018](#_ENREF_15)) |
| sarJ | Insulator | AGACTGTCGCCGGATGTGTATCCGACCTGACGATGGCCCAAAAGGGCCGAAACAGTCCTCTACAAATAATTTTGTTTAA | ([Lou et al, 2012](#_ENREF_16)) |
| riboJ01 | Insulator | AGCGCTCAACGGGTGTGCTTCCCGTTCTGATGAGTCCGTGAGGACGAAAGCGCCTCTACAAATAATTTTGTTTAA | ([Nielsen et al, 2016](#_ENREF_19)) |
| HH2 | Ribozyme* | NNNNNNNCTGATGAGTCCGTGAGGACGAAACGAGCTAGCTCGTCNNNNNN | ([Ghodasara & Voigt, 2017](#_ENREF_10)) |
| HH3 | Ribozyme* | NNNNNNNCTGATGAGGTATGACCCACGAAACGTCCCTCTTCGTCNNNNNN | ([Ghodasara & Voigt, 2017](#_ENREF_10)) |
| sRNA(*pta*) | sRNA | GCCGATTTGGCGGGT | This work |
| sRNA(*rfp*) | sRNA | GTCAAAGTTTGTTAT | ([Ghodasara & Voigt, 2017](#_ENREF_10)) |
| Spot42-1776 | sRNA scaffold | ATTTGTAGAAATATTTTATTCGCCCCCGGAAGATCATTCCGGGGGCTTTTTTATT | ([Ghodasara & Voigt, 2017](#_ENREF_10)) |
| sRNA barcode | sRNA | ATAACAAACTTTGAC | ([Ghodasara & Voigt, 2017](#_ENREF_10)) |
| sgRNA(*rfp*) | sgRNA | AACTTTCAGTTTAGCGGTCTGTTTTAGAGCTAGAAATAGCAAGTTAAAATAAGGCTAGTCCGTTATCAACTTGAAAAAGTGGCACCGAGTCGGTGCTTTTTTT | ([Qi et al, 2013](#_ENREF_22)) |
| sgRNA(*pta*) | sgRNA | TTCGTTACCGCCGATTTGGCGTTTTAGAGCTAGAAATAGCAAGTTAAAATAAGGCTAGTCCGTTATCAACTTGAAAAAGTGGCACCGAGTCGGTGCTTTTTTT | This work |
| sgRNA(*poxB*) | sgRNA | CCATCTCCTGAATGTGATAAGTTTTAGAGCTAGAAATAGCAAGTTAAAATAAGGCTAGTCCGTTATCAACTTGAAAAAGTGGCACCGAGTCGGTGCTTTTTTT | This work |
| FLP recombinase | scar site | GAAGTTCCTATACTTTCTAGAGAATAGGAACTTCGGAATAGGAACT | ([Datsenko & Wanner, 2000](#_ENREF_6)) |
| BBa_B0032 | RBS | TCACACAGGAAAG | ([2016](#_ENREF_1)) |
| BBa_B0034 | RBS | AAAGAGGAGAAA | ([2016](#_ENREF_1)) |
| RBS_D9 | RBS | ACGGTTCGAAAGGGAGACATAATAG | This work |
| RBS_F5 | RBS | AGTAGCCTTCGAGTAGGAGACTCGTTCTAG | This work |
| RBS_H4 | RBS | AAGACTACCCTTTCAATCAGGGATTCCCAGG | This work |
| *mRFP1* | CDS | ATGGCTTCCTCCGAAGACGTTATCAAAGAGTTCATGCGTTTCAAAGTTCGTATGGAAGGTTCCGTTAACGGTCACGAGTTCGAAATCGAAGGTGAAGGTGAAGGTCGTCCGTACGAAGGTACCCAGACCGCTAAACTGAAAGTTACCAAAGGTGGTCCGCTGCCGTTCGCTTGGGACATCCTGTCCCCGCAGTTCCAGTACGGTTCCAAAGCTTACGTTAAACACCCGGCTGACATCCCGGACTACCTGAAACTGTCCTTCCCGGAAGGTTTCAAATGGGAACGTGTTATGAACTTCGAAGACGGTGGTGTTGTTACCGTTACCCAGGACTCCTCCCTGCAAGACGGTGAGTTCATCTACAAAGTTAAACTGCGTGGTACCAACTTCCCGTCCGACGGTCCGGTTATGCAGAAAAAAACCATGGGTTGGGAAGCTTCCACCGAACGTATGTACCCGGAAGACGGTGCTCTGAAAGGTGAAATCAAAATGCGTCTGAAACTGAAAGACGGTGGTCACTACGACGCTGAAGTTAAAACCACCTACATGGCTAAAAAACCGGTTCAGCTGCCGGGTGCTTACAAAACCGACATCAAACTGGACATCACCTCCCACAACGAAGACTACACCATCGTTGAACAGTACGAACGTGCTGAAGGTCGTCACTCCACCGGTGCTTAATAA | ([Qi et al, 2013](#_ENREF_22)) |
| *sfGFP* | CDS | ATGCGTAAAGGCGAAGAGCTGTTCACTGGTGTCGTCCCTATTCTGGTGGAACTGGATGGTGATGTCAACGGTCATAAGTTTTCCGTGCGTGGCGAGGGTGAAGGTGACGCAACTAATGGTAAACTGACGCTGAAGTTCATCTGTACTACTGGTAAACTGCCGGTACCTTGGCCGACTCTGGTAACGACGCTGACTTATGGTGTTCAGTGCTTTGCTCGTTATCCGGACCATATGAAGCAGCATGACTTCTTCAAGTCCGCCATGCCGGAAGGCTATGTGCAGGAACGCACGATTTCCTTTAAGGATGACGGCACGTACAAAACGCGTGCGGAAGTGAAATTTGAAGGCGATACCCTGGTAAACCGCATTGAGCTGAAAGGCATTGACTTTAAAGAAGACGGCAATATCCTGGGCCATAAGCTGGAATACAATTTTAACAGCCACAATGTTTACATCACCGCCGATAAACAAAAAAATGGCATTAAAGCGAATTTTAAAATTCGCCACAACGTGGAGGATGGCAGCGTGCAGCTGGCTGATCACTACCAGCAAAACACTCCAATCGGTGATGGTCCTGTTCTGCTGCCAGACAATCACTATCTGAGCACGCAAAGCGTTCTGTCTAAAGATCCGAACGAGAAACGCGATCATATGGTTCTGCTGGAGTTCGTAACCGCAGCGGGCATCACGCATGGTATGGATGAACTGTACAAATGATAA | ([Segall-Shapiro et al, 2014](#_ENREF_26)) |
| *PhlF* | CDS | ATGGCACGTACCCCGAGCCGTAGCAGCATTGGTAGCCTGCGTAGTCCGCATACCCATAAAGCAATTCTGACCAGCACCATTGAAATCCTGAAAGAATGTGGTTATAGCGGTCTGAGCATTGAAAGCGTTGCACGTCGTGCCGGTGCAAGCAAACCGACCATTTATCGTTGGTGGACCAATAAAGCAGCACTGATTGCCGAAGTGTATGAAAATGAAAGCGAACAGGTGCGTAAATTTCCGGATCTGGGTAGCTTTAAAGCCGATCTGGATTTTCTGCTGCGTAATCTGTGGAAAGTTTGGCGTGAAACCATTTGTGGTGAAGCATTTCGTTGTGTTATTGCAGAAGCACAGCTGGACCCTGCAACCCTGACCCAGCTGAAAGATCAGTTTATGGAACGTCGTCGTGAGATGCCGAAAAAACTGGTTGAAAATGCCATTAGCAATGGTGAACTGCCGAAAGATACCAATCGTGAACTGCTGCTGGATATGATTTTTGGTTTTTGTTGGTATCGCCTGCTGACCGAACAGCTGACCGTTGAACAGGATATTGAAGAATTTACCTTCCTGCTGATTAATGGTGTTTGTCCGGGTACACAGCGTTAA | ([Stanton et al, 2014b](#_ENREF_28)) |
| *TetR* | CDS | ATGTCCAGATTAGATAAAAGTAAAGTGATTAACAGCGCATTAGAGCTGCTTAATGAGGTCGGAATCGAAGGTTTAACAACCCGTAAACTCGCCCAGAAGCTAGGTGTAGAGCAGCCTACATTGTATTGGCATGTAAAAAATAAGCGGGCTTTGCTCGACGCCTTAGCCATTGAGATGTTAGATAGGCACCATACTCACTTTTGCCCTTTAGAAGGGGAAAGCTGGCAAGATTTTTTACGTAATAACGCTAAAAGTTTTAGATGTGCTTTACTAAGTCATCGCGATGGAGCAAAAGTACATTTAGGTACACGGCCTACAGAAAAACAGTATGAAACTCTCGAAAATCAATTAGCCTTTTTATGCCAACAAGGTTTTTCACTAGAGAATGCATTATATGCACTCAGCGCTGTGGGGCATTTTACTTTAGGTTGCGTATTGGAAGATCAAGAGCATCAAGTCGCTAAAGAAGAAAGGGAAACACCTACTACTGATAGTATGCCGCCATTATTACGACAAGCTATCGAATTATTTGATCACCAAGGTGCAGAGCCAGCCTTCTTATTCGGCCTTGAATTGATCATATGCGGATTAGAAAAACAACTTAAATGTGAAAGTGGGTCCTAA | Registry of Biological Parts |
| *sicA* | CDS | ATGGATTATCAAAATAATGTCAGCGAAGAACGTGTTGCGGAAATGATTTGGGATGCCGTTAGTGAAGGCGCCACGCTAAAAGACGTTCATGGGATCCCTCAAGATATGATGGACGGTTTATATGCTCATGCTTATGAGTTTTATAACCAGGGACGACTGGATGAAGCTGAGACGTTCTTTCGTTACTTATGCATTTATGATTTTTACAATCCCGATTACACCATGGGACTGGCGGCAGTATGCCAACTGAAAAAACAATTTCAGAAAGCATGTGACCTTTATGCAGTAGCGTTTACGTTACTTAAAAATGATTATCGCCCCGTTTTTTTTACCGGGCAGTGTCAATTATTAATGCGTAAGGCAGCAAAAGCCAGACAGTGTTTTGAACTTGTCAATGAACGTACTGAAGATGAGTCTCTGCGGGCAAAAGCGTTGGTCTATCTGGAGGCGCTAAAAACGGCGGAGACAGAGCAGCACAGTGAACAAGAAAAGGAATAA | ([Moon et al, 2012](#_ENREF_17)) |
| *invF* | CDS | ATGTTGAACACCCAAGAGGTTTTGAAGGAGGGTGAAAAAAGAAAGATAAGATCACCTGAGGCTTGGTTCATCCAAACCTGCAGTGCTCAGAAATTACACATGAGCTTCAGCGAGTCAAGGCATAACGAGAACTGTTTAATACAAGAGGGGGCTTTATTGTTCTGTGAACAAGCAGTAGTAGCTCCTGTTAGCGGTGATTTAGTATTCAGGCCTCTCAAGATAGAGGTTTTGTCAAAGCTCTTAGCTTTCATAGACGGGGCTGGCCTCGTTGATACCACCTACGCAGAGAGTGACAAGTGGGTACTCTTATCACCGGAATTCAGAGCAATATGGCAGGACAGAAAGAGATGTGAATATTGGTTCCTCCAACAGATAATAACCCCGAGCCCTGCATTTAACAAAGTTTTAGCTTTACTCAGGAAGTCAGAATCATATTGGCTCGTAGGGTACCTCTTGGCACAAAGCACGTCAGGGAATACCATGCGTATGTTAGGTGAGGATTACGGGGTAAGCTACACGCACTTCCGCCGTCTCTGTTCACGTGCTCTCGGGGGTAAGGCTAAATCAGAGCTCAGGAATTGGAGAATGGCTCAGAGCTTATTAAACTCAGTTGAGGGGCATGAAAATATAACGCAGCTCGCAGTAAACCACGGGTATAGCAGCCCGAGCCACTTCAGCTCAGAAATAAAGGAATTAATAGGGGTAAGCCCTAGAAAGCTCAGCAACATAATACAGCTCGCTGATAAGTGA | ([Moon et al, 2012](#_ENREF_17)) |
| *dCas9* | CDS | ATGGATAAGAAATACTCAATAGGCTTAGCTATCGGCACAAATAGCGTCGGATGGGCGGTGATCACTGATGAATATAAGGTTCCGTCTAAAAAGTTCAAGGTTCTGGGAAATACAGACCGCCACAGTATCAAAAAAAATCTTATAGGGGCTCTTTTATTTGACAGTGGAGAGACAGCGGAAGCGACTCGTCTCAAACGGACAGCTCGTAGAAGGTATACACGTCGGAAGAATCGTATTTGTTATCTACAGGAGATTTTTTCAAATGAGATGGCGAAAGTAGATGATAGTTTCTTTCATCGACTTGAAGAGTCTTTTTTGGTGGAAGAAGACAAGAAGCATGAACGTCATCCTATTTTTGGAAATATAGTAGATGAAGTTGCTTATCATGAGAAATATCCAACTATCTATCATCTGCGAAAAAAATTGGTAGATTCTACTGATAAAGCGGATTTGCGCTTAATCTATTTGGCCTTAGCGCATATGATTAAGTTTCGTGGTCATTTTTTGATTGAGGGAGATTTAAATCCTGATAATAGTGATGTGGACAAACTATTTATCCAGTTGGTACAAACCTACAATCAATTATTTGAAGAAAACCCTATTAACGCAAGTGGAGTAGATGCTAAAGCGATTCTTTCTGCACGATTGAGTAAATCAAGACGATTAGAAAATCTCATTGCTCAGCTCCCCGGTGAGAAGAAAAATGGCTTATTTGGGAATCTCATTGCTTTGTCATTGGGTTTGACCCCTAATTTTAAATCAAATTTTGATTTGGCAGAAGATGCTAAATTACAGCTTTCAAAAGATACTTACGATGATGATTTAGATAATTTATTGGCGCAAATTGGAGATCAATATGCTGATTTGTTTTTGGCAGCTAAGAATTTATCAGATGCTATTTTACTTTCAGATATCCTAAGAGTAAATACTGAAATAACTAAGGCTCCCCTATCAGCTTCAATGATTAAACGCTACGATGAACATCATCAAGACTTGACTCTTTTAAAAGCTTTAGTTCGACAACAACTTCCAGAAAAGTATAAAGAAATCTTTTTTGATCAATCAAAAAACGGATATGCAGGTTATATTGATGGGGGAGCTAGCCAAGAAGAATTTTATAAATTTATCAAACCAATTTTAGAAAAAATGGATGGTACTGAGGAATTATTGGTGAAACTAAATCGTGAAGATTTGCTGCGCAAGCAACGGACCTTTGACAACGGCTCTATTCCCCATCAAATTCACTTGGGTGAGCTGCATGCTATTTTGAGAAGACAAGAAGACTTTTATCCATTTTTAAAAGACAATCGTGAGAAGATTGAAAAAATCTTGACTTTTCGAATTCCTTATTATGTTGGTCCATTGGCGCGTGGCAATAGTCGTTTTGCATGGATGACTCGGAAGTCTGAAGAAACAATTACCCCATGGAATTTTGAAGAAGTTGTCGATAAAGGTGCTTCAGCTCAATCATTTATTGAACGCATGACAAACTTTGATAAAAATCTTCCAAATGAAAAAGTACTACCAAAACATAGTTTGCTTTATGAGTATTTTACGGTTTATAACGAATTGACAAAGGTCAAATATGTTACTGAAGGAATGCGAAAACCAGCATTTCTTTCAGGTGAACAGAAGAAAGCCATTGTTGATTTACTCTTCAAAACAAATCGAAAAGTAACCGTTAAGCAATTAAAAGAAGATTATTTCAAAAAAATAGAATGTTTTGATAGTGTTGAAATTTCAGGAGTTGAAGATAGATTTAATGCTTCATTAGGTACCTACCATGATTTGCTAAAAATTATTAAAGATAAAGATTTTTTGGATAATGAAGAAAATGAAGATATCTTAGAGGATATTGTTTTAACATTGACCTTATTTGAAGATAGGGAGATGATTGAGGAAAGACTTAAAACATATGCTCACCTCTTTGATGATAAGGTGATGAAACAGCTTAAACGTCGCCGTTATACTGGTTGGGGACGTTTGTCTCGAAAATTGATTAATGGTATTAGGGATAAGCAATCTGGCAAAACAATATTAGATTTTTTGAAATCAGATGGTTTTGCCAATCGCAATTTTATGCAGCTGATCCATGATGATAGTTTGACATTTAAAGAAGACATTCAAAAAGCACAAGTGTCTGGACAAGGCGATAGTTTACATGAACATATTGCAAATTTAGCTGGTAGCCCTGCTATTAAAAAAGGTATTTTACAGACTGTAAAAGTTGTTGATGAATTGGTCAAAGTAATGGGGCGGCATAAGCCAGAAAATATCGTTATTGAAATGGCACGTGAAAATCAGACAACTCAAAAGGGCCAGAAAAATTCGCGAGAGCGTATGAAACGAATCGAAGAAGGTATCAAAGAATTAGGAAGTCAGATTCTTAAAGAGCATCCTGTTGAAAATACTCAATTGCAAAATGAAAAGCTCTATCTCTATTATCTCCAAAATGGAAGAGACATGTATGTGGACCAAGAATTAGATATTAATCGTTTAAGTGATTATGATGTCGATGCCATTGTTCCACAAAGTTTCCTTAAAGACGATTCAATAGACAATAAGGTCTTAACGCGTTCTGATAAAAATCGTGGTAAATCGGATAACGTTCCAAGTGAAGAAGTAGTCAAAAAGATGAAAAACTATTGGAGACAACTTCTAAACGCCAAGTTAATCACTCAACGTAAGTTTGATAATTTAACGAAAGCTGAACGTGGAGGTTTGAGTGAACTTGATAAAGCTGGTTTTATCAAACGCCAATTGGTTGAAACTCGCCAAATCACTAAGCATGTGGCACAAATTTTGGATAGTCGCATGAATACTAAATACGATGAAAATGATAAACTTATTCGAGAGGTTAAAGTGATTACCTTAAAATCTAAATTAGTTTCTGACTTCCGAAAAGATTTCCAATTCTATAAAGTACGTGAGATTAACAATTACCATCATGCCCATGATGCGTATCTAAATGCCGTCGTTGGAACTGCTTTGATTAAGAAATATCCAAAACTTGAATCGGAGTTTGTCTATGGTGATTATAAAGTTTATGATGTTCGTAAAATGATTGCTAAGTCTGAGCAAGAAATAGGCAAAGCAACCGCAAAATATTTCTTTTACTCTAATATCATGAACTTCTTCAAAACAGAAATTACACTTGCAAATGGAGAGATTCGCAAACGCCCTCTAATCGAAACTAATGGGGAAACTGGAGAAATTGTCTGGGATAAAGGGCGAGATTTTGCCACAGTGCGCAAAGTATTGTCCATGCCCCAAGTCAATATTGTCAAGAAAACAGAAGTACAGACAGGCGGATTCTCCAAGGAGTCAATTTTACCAAAAAGAAATTCGGACAAGCTTATTGCTCGTAAAAAAGACTGGGATCCAAAAAAATATGGTGGTTTTGATAGTCCAACGGTAGCTTATTCAGTCCTAGTGGTTGCTAAGGTGGAAAAAGGGAAATCGAAGAAGTTAAAATCCGTTAAAGAGTTACTAGGGATCACAATTATGGAAAGAAGTTCCTTTGAAAAAAATCCGATTGACTTTTTAGAAGCTAAAGGATATAAGGAAGTTAAAAAAGACTTAATCATTAAACTACCTAAATATAGTCTTTTTGAGTTAGAAAACGGTCGTAAACGGATGCTGGCTAGTGCCGGAGAATTACAAAAAGGAAATGAGCTGGCTCTGCCAAGCAAATATGTGAATTTTTTATATTTAGCTAGTCATTATGAAAAGTTGAAGGGTAGTCCAGAAGATAACGAACAAAAACAATTGTTTGTGGAGCAGCATAAGCATTATTTAGATGAGATTATTGAGCAAATCAGTGAATTTTCTAAGCGTGTTATTTTAGCAGATGCCAATTTAGATAAAGTTCTTAGTGCATATAACAAACATAGAGACAAACCAATACGTGAACAAGCAGAAAATATTATTCATTTATTTACGTTGACGAATCTTGGAGCTCCCGCTGCTTTTAAATATTTTGATACAACAATTGATCGTAAACGATATACGTCTACAAAAGAAGTTTTAGATGCCACTCTTATCCATCAATCCATCACTGGTCTTTATGAAACACGCATTGATTTGAGTCAGCTAGGAGGTGACTAA | ([Nielsen & Voigt, 2014](#_ENREF_20); [Qi et al, 2013](#_ENREF_22)) |
| *mf-LON* | CDS | ATGAGTAAAAAAATCAAACTACCTATTTTCCAAATCCGTGGTTCTTTCATCGTTCCGGGTATCAAAGAAAACCTGGAAGTTGGTCGTAAAAACACCCTGGCTTCTGTTAACTACGCTATCAAAAACTCTAACAACCAGATGATCGCTATCCCGCAGATCGACGCTTCTGTTGAAAAACCGGAGTTCTCTGACCTGCACGAGTTCGGTATCCTGATCGACTTCGAAGTTATCAAAGAATGGAAAGACAACTCTCTGACCATCTCTACCAACCCGATCCAGCGTTGCAAAGTTATCTCTTTCTTCGAAAACGAAGACCAGGTTCCGTACGCTGAAGTTGAACTGATCGAATCTATCAACGACTTCTCTGACGAAGAACTGAAAGAACTGATCGAAAAAATCTCTGACGCTATCAAAACCAAGGCTTCTCTGGTTACCAAACAGATCAAACAACTGATCTCTGGTGAATCTGACGACCTGTCTCTGGCTTTCGACTCTATCATGTTCAAACTGGCTCCGTCTAAAATCCTGACCAACCCGGAATACATCACCTCTCCGTCTCTGAAAACCCGTTGGTCTATCATCGAAAAAATCATCTTCGCTGAAGACGGTATCATCACCCGTAACGCTGAATCTATCGACGCTGCTCGTCAGAAAAACGAAATCGAACAGGAACTGAACCACAAACTGAAAGAAAAAATGGACAAACAGCAGAAAGAATACTACCTGCGTGAAAAAATGCGTATCATCAAAGACGAACTGGAAGACGAAGACGACTCTGACGACTCTTCTCTGGAAAAATACAAAGAACGTCTGGCTAAAGAACCGTTCCCGGAAGAAGTTAAACGTAAAATCATGGCTTCTATCAAACGTGTTGAAGCTCTGCAATCTGGAACCCCGGAATGGAACACCGAAAAAAACTACATCGACTGGATGATGTCTATCCCGTGGTGGGAAGAAACCGAAGACCTGACCGACCTGAAATACGCTAAAAAAATCCTGGACAAACACCACTACGGTATGAAAAAAGTTAAAGAACGTATCATCGAATACCTGGCTGTTAAAACCAAAACCAAATCTCTGAAAGCTCCGATCATCACCCTGGTTGGTCCGCCGGGTGTTGGTAAAACCTCTCTGGCTAAATCTATCGCTGAAGCTGTTGGTAAAAACTTCGTTAAAGTTTCTCTGGGTGGTGTTAAAGACGAATCTGAAATCCGTGGTCACCGTAAAACCTACGTTGGTTCTATGCCGGGTCGTATCATCCAGACCATGAAACGTGCTAAAGTTAAAAACCCGCTGTTCCTGCTGGACGAAATCGACAAAATGGCTTCTGACCACCGTGGTGACCCGGCTTCTGCTATGCTGGAAGTTCTGGACCCGGAACAGAACAAAGAGTTCTCTGACCACTACATCGAAGAACCGTACGACCTGTCTCAGGTTATGTTCATCGCTACCGCTAACTACCCGGAAGACATCCCGGAAGCTCTGTACGACCGTATGGAAATCATCAACCTGTCTTCTTACACCGAAATCGAAAAAGTTAAAATCGCTCAGGACTACCTGGTTCCGAAAGCTATCGAACAGCACGAACTGACCTCTGAAGAAATCTCTTTCACCGAAGGTGCTATCAACGAAATCATCAAATACTACACCCGTGAAGCTGGTGTTCGTCAACTGGAACGTCACATCAACTCTATCATCCGTAAATACATCGTTAAAAACCTGAACGGTGAAATGGACAAAATCGTTATCGACGAAAAACAGGTTAACGACCTGCTGGGTAAACGTATCTTCGACCACACCGAAAAACAGGAAGAATCTCAGATCGGTGTTGTTACCGGTCTGGCTTACACCCAGTTCGGTGGTGACATCCTGCCGATTGAAGTTTCTCTGTATCCGGGTAAAGGTAACCTGATCCTGACCGGTAAACTGGGTGAAGTTATGAAAGAATCTGCTACCATCGCTCTGACCTACGTTAAATCTAACTTCGAAAAATTCGGTGTTGACAAAAAAGTTTTCGAAGAAAACGACATCCACGTTCACGTTCCGGAAGGTGCTGTTCCGAAAGACGGTCCGTCTGCTGGTATCACCATCACCACCGCTCTGATCTCTGCTCTTTCTGACAAACCGGTTTCTAAAGAAATCGGTATGACCGGTGAAATCACCCTGCGTGGTAACGTTCTGCCGATTGGTGGTCTGCGTGAAAAATCTATCTCTGCTTCTCGTTCTGGTCTGAAAACCATCATCATCCCGAAAAAAAACGAACGTGACCTGGACGAAATCCCGGACGAAGTTAAAGCTAAACTGAAAATCATCCCGGCTGAAAAATACGAAGAGGTATTTGCAATAGTTTTTAAAACAAAAGCGGCGAACAAAAACGAAGAAAACACCAACGAAGTGCCGGACGGTATGCTGAACGCGGGCCAGGCGAACAGAAGACGAGTTTAA | ([Cameron & Collins, 2014](#_ENREF_3)) |
| *(MBP) -SuMMV* | CDS | ATGAAAATCGAAGAAGGTAAACTGGTAATCTGGATTAACGGCGATAAGGGCTATAACGGTCTCGCTGAAGTCGGTAAGAAATTCGAGAAAGATACCGGAATTAAAGTCACCGTTGAGCATCCGGATAAACTGGAAGAGAAATTCCCACAGGTTGCGGCAACTGGCGATGGCCCTGACATTATCTTCTGGGCACACGACCGCTTTGGTGGCTACGCTCAATCTGGCCTGTTGGCTGAAATCACCCCGGACAAAGCGTTCCAGGACAAGCTGTATCCGTTTACCTGGGATGCCGTACGTTACAACGGCAAGCTGATTGCTTACCCGATCGCTGTTGAAGCGTTATCGCTGATTTATAACAAAGATCTGCTGCCGAACCCGCCAAAAACCTGGGAAGAGATCCCGGCGCTGGATAAAGAACTGAAAGCGAAAGGTAAGAGCGCGCTGATGTTCAACCTGCAAGAACCGTACTTCACCTGGCCGCTGATTGCTGCTGACGGGGGTTATGCGTTCAAGTATGAAAACGGCAAGTACGACATTAAAGACGTGGGCGTGGATAACGCTGGCGCGAAAGCGGGTCTGACCTTCCTGGTTGACCTGATTAAAAACAAACACATGAATGCAGACACCGATTACTCCATCGCAGAAGCTGCCTTTAATAAAGGCGAAACAGCGATGACCATCAACGGCCCGTGGGCATGGTCCAACATCGACACCAGCAAAGTGAATTATGGTGTAACGGTACTGCCGACCTTCAAGGGTCAACCATCCAAACCGTTCGTTGGCGTGCTGAGCGCAGGTATTAACGCCGCCAGTCCGAACAAAGAGCTGGCGAAAGAGTTCCTCGAAAACTATCTGCTGACTGATGAAGGTCTGGAAGCGGTTAATAAAGACAAACCGCTGGGTGCCGTAGCGCTGAAGTCTTACGAGGAAGAGTTGGCGAAAGATCCACGTATTGCCGCCACCATGGAAAACGCCCAGAAAGGTGAAATCATGCCGAACATCCCGCAGATGTCCGCTTTCTGGTATGCCGTGCGTACTGCGGTGATCAACGCCGCCAGCGGTCGTCAGACTGTCGATGAAGCCCTGAAAGACGCGCAGACTCGTATCACCAAGGGCGGTAGCGGTGAAGAAATTCATCTGCAGGGTCATCACCACCATCATCACGGTGTGTCTCTGAGTCGTGGTGTGCGTGACTATAACGCAATTAGTAGCATGGTTTGTCGCGTGACCAACGATTCCGGTTCTAGCTCTACCACCATGTACGGTATTGGTTACGGCTGTTATATCATCACCAACAAACACTTGTTCCGTGAAAATAACGGCCGTCTGCTTATCACTTCTCACCACGGCGAATACATTTGCAAAAATTCCGCGTCTCTTAAACTGTCTCTGGTTCCGGGTCGCGATATGCTGCTGATCCGTCTTCCGAAAGATTGCCCACCGTTCCCGAGCAAACTCAAGTTCCGCGAACCGACGAGCGAAGAAAAAGCGGTGCTTGTTGTTACCAACTTTCAGGAAAAACACCTGTCTTCTATGGTTTCGGAAAGCAGCTGCGTTGTACAGCGTGAAGACTCTCCGATTTGGCGTCATTGGATCTCCACCAAAGATGGTCATTGTGGCGCCCCGATCGTATCTATTCGTGACGGTTACATCATTGGTTCTCACTGCGGTGAGAACCCGATGACTAGCAACTTCTTCACCAGCATCCCTAAAGATTTCCAGAACCTGCTGAATGGTAAAGAAGCGAACGAGTGGGTTTCCGGTTGGAAGTACAACATCGACGCGGTATGTTGGGGTGGCCTGAGCGTTGTTAACGACGCGCCGAGCGAACCATTCATCACCGCAAAAGTTGTGAGCGCCCTGGACACCGAGGGTATCAAAGTCCAGTAA | ([Fernandez-Rodriguez & Voigt, 2016](#_ENREF_8)) |
| *poxB* | CDS | ATGAAACAAACGGTTGCAGCTTATATCGCCAAAACACTCGAATCGGCAGGGGTGAAACGCATCTGGGGAGTCACAGGCGACTCTCTGAACGGTCTTAGTGACAGTCTTAATCGCATGGGCACCATCGAGTGGATGTCCACCCGCCACGAAGAAGTGGCGGCCTTTGCCGCTGGCGCTGAAGCACAACTTAGCGGAGAACTGGCGGTCTGCGCCGGATCGTGCGGCCCCGGCAACCTGCACTTAATCAACGGCCTGTTCGATTGCCACCGCAATCACGTTCCGGTACTGGCGATTGCCGCTCATATTCCCTCCAGCGAAATTGGCAGCGGCTATTTCCAGGAAACCCACCCACAAGAGCTATTCCGCGAATGTAGTCACTATTGCGAGCTGGTTTCCAGCCCGGAGCAGATCCCACAAGTACTGGCGATTGCCATGCGCAAAGCGGTGCTTAACCGTGGCGTTTCGGTTGTCGTGTTACCAGGCGACGTGGCGTTAAAACCTGCGCCAGAAGGGGCAACCATGCACTGGTATCATGCGCCACAACCAGTCGTGACGCCGGAAGAAGAAGAGTTACGCAAACTGGCGCAACTGCTGCGTTATTCCAGCAATATCGCCCTGATGTGTGGCAGCGGCTGCGCGGGGGCGCATAAAGAGTTAGTTGAGTTTGCCGGGAAAATTAAAGCGCCTATTGTTCATGCCCTGCGCGGTAAAGAACATGTCGAATACGATAATCCGTATGATGTTGGAATGACCGGGTTAATCGGCTTCTCGTCAGGTTTCCATACCATGATGAACGCCGACACGTTAGTGCTACTCGGCACGCAATTTCCCTACCGCGCCTTCTACCCGACCGATGCCAAAATCATTCAGATTGATATCAACCCAGCCAGCATCGGCGCTCACAGCAAGGTGGATATGGCACTGGTCGGCGATATCAAGTCGACTCTGCGTGCATTGCTTCCATTGGTGGAAGAAAAAGCCGATCGCAAGTTTCTGGATAAAGCGCTGGAAGATTACCGCGACGCCCGCAAAGGGCTGGACGATTTAGCTAAACCGAGCGAGAAAGCCATTCACCCGCAATATCTGGCGCAGCAAATTAGTCATTTTGCCGCCGATGACGCTATTTTCACCTGTGACGTTGGTACGCCAACGGTGTGGGCGGCACGTTATCTAAAAATGAACGGCAAGCGTCGCCTGTTAGGTTCGTTTAACCACGGTTCGATGGCTAACGCCATGCCGCAGGCGCTGGGTGCGCAGGCGACAGAGCCAGAACGTCAGGTGGTCGCCATGTGCGGCGATGGCGGTTTTAGCATGTTGATGGGCGATTTCCTCTCAGTAGTGCAGATGAAACTGCCAGTGAAAATTGTCGTCTTTAACAACAGCGTGCTGGGCTTTGTGGCGATGGAGATGAAAGCTGGTGGCTATTTGACTGACGGCACCGAACTACACGACACAAACTTTGCCCGCATTGCCGAAGCGTGCGGCATTACGGGTATCCGTGTAGAAAAAGCGTCTGAAGTTGATGAAGCCCTGCAACGCGCCTTCTCCATCGACGGTCCGGTGTTGGTGGATGTGGTGGTCGCCAAAGAAGAGTTAGCCATTCCACCGCAGATCAAACTCGAACAGGCCAAAGGTTTCAGCCTGTATATGCTGCGCGCAATCATCAGCGGACGCGGTGATGAAGTGATCGAACTGGCGAAAACAAACTGGCTAAGGTAA | *E. coli* genome |
| *poxB-E170* | CDS | ATGAAACAAACGGTTGCAGCTTATATCGCCAAAACACTCGAATCGGCAGGGGTGAAACGCATCTGGGGAGTCACAGGCGACTCTCTGAACGGTCTTAGTGACAGTCTTAATCGCATGGGCACCATCGAGTGGATGTCCACCCGCCACGAAGAAGTGGCGGCCTTTGCCGCTGGCGCTGAAGCACAACTTAGCGGAGAACTGGCGGTCTGCGCCGGATCGTGCGGCCCCGGCAACCTGCACTTAATCAACGGCCTGTTCGATTGCCACCGCAATCACGTTCCGGTACTGGCGATTGCCGCTCATATTCCCTCCAGCGAAATTGGCAGCGGCTATTTCCAGGAAACCCACCCACAAGAGCTATTCCGCGAATGTAGTCACTATTGCGAGCTGGTTTCCAGCCCGGAGCAGATCCCACAAGTACTGGCGATTGCCATGCGCAAAGCGGTGCTTAACCGTGGCGTTTCGGTTGTCGTGTTACCAGGCGACGTGGCGTTAAAACCTGCGCCA**GAAGAAATTCATCTGCAGTTCTTATTCGTGCAA**CAACCAGTCGTGACGCCGGAAGAAGAAGAGTTACGCAAACTGGCGCAACTGCTGCGTTATTCCAGCAATATCGCCCTGATGTGTGGCAGCGGCTGCGCGGGGGCGCATAAAGAGTTAGTTGAGTTTGCCGGGAAAATTAAAGCGCCTATTGTTCATGCCCTGCGCGGTAAAGAACATGTCGAATACGATAATCCGTATGATGTTGGAATGACCGGGTTAATCGGCTTCTCGTCAGGTTTCCATACCATGATGAACGCCGACACGTTAGTGCTACTCGGCACGCAATTTCCCTACCGCGCCTTCTACCCGACCGATGCCAAAATCATTCAGATTGATATCAACCCAGCCAGCATCGGCGCTCACAGCAAGGTGGATATGGCACTGGTCGGCGATATCAAGTCGACTCTGCGTGCATTGCTTCCATTGGTGGAAGAAAAAGCCGATCGCAAGTTTCTGGATAAAGCGCTGGAAGATTACCGCGACGCCCGCAAAGGGCTGGACGATTTAGCTAAACCGAGCGAGAAAGCCATTCACCCGCAATATCTGGCGCAGCAAATTAGTCATTTTGCCGCCGATGACGCTATTTTCACCTGTGACGTTGGTACGCCAACGGTGTGGGCGGCACGTTATCTAAAAATGAACGGCAAGCGTCGCCTGTTAGGTTCGTTTAACCACGGTTCGATGGCTAACGCCATGCCGCAGGCGCTGGGTGCGCAGGCGACAGAGCCAGAACGTCAGGTGGTCGCCATGTGCGGCGATGGCGGTTTTAGCATGTTGATGGGCGATTTCCTCTCAGTAGTGCAGATGAAACTGCCAGTGAAAATTGTCGTCTTTAACAACAGCGTGCTGGGCTTTGTGGCGATGGAGATGAAAGCTGGTGGCTATTTGACTGACGGCACCGAACTACACGACACAAACTTTGCCCGCATTGCCGAAGCGTGCGGCATTACGGGTATCCGTGTAGAAAAAGCGTCTGAAGTTGATGAAGCCCTGCAACGCGCCTTCTCCATCGACGGTCCGGTGTTGGTGGATGTGGTGGTCGCCAAAGAAGAGTTAGCCATTCCACCGCAGATCAAACTCGAACAGGCCAAAGGTTTCAGCCTGTATATGCTGCGCGCAATCATCAGCGGACGCGGTGATGAAGTGATCGAACTGGCGAAAACAAACTGGCTAAGGTAA | This work |
| ASV tag | tag | GCAGCAAACGACGAAAACTACGCTGCTTCTGTT | This work |
| pdt#3E | tag | GCGGCGAACAAAAACGAAGAAAACACCAACGAAGTGCCGGACGGTATGCTGAACGCGGGCCAGGCGAACAGAAGACGAGTT | ([Cameron & Collins, 2014](#_ENREF_3)) |
| SuMMV tag (EEIHLQ-FLFVQ) | tag | GAAGAAATTCATCTGCAGTTCTTATTCGTGCAA | ([Fernandez-Rodriguez & Voigt, 2016](#_ENREF_8)) |
| BBa_B0015 | Terminator | CCAGGCATCAAATAAAACGAAAGGCTCAGTCGAAAGACTGGGCCTTTCGTTTTATCTGTTGTTTGTCGGTGAACGCTCTCTACTAGAGTCACACTGGCTCACCTTCGGGTGGGCCTTTCTGCGTTTAT | ([2016](#_ENREF_1)) |
| DT5 | Terminator | AAAAAAGCGGCTAACCACGCCGCTTTTTTTACGTCTGCATCCTTGGCCCTCCATCCTTAGATAGCTCGGTACCAAATTCCAGAAAAGAGGCCTCCCGAAAGGGGGGCCTTTTTTCGTTTTGGTCCTCATAGGCAATACGATCGCATGTCC | ([Chen et al, 2013](#_ENREF_4)) |
| DT11 | Terminator | AACGCATGAGAAAGCCCCCGGAAGATCACCTTCCGGGGGCTTTTTTATTGCGCTCCTTGGCCCTCCATCCTTAGATAGCTCGGTACCAAATTCCAGAAAAGAGGCCTCCCGAAAGGGGGGCCTTTTTTCGTTTTGGTCC | ([Chen et al, 2013](#_ENREF_4)) |
| DT14 | Terminator | TTCAGCCAAAAAACTTAAGACCGCCGGTCTTGTCCACTACCTTGCAGTAATGCGGTGGACAGGATCGGCGGTTTTCTTTTCTCTTCTCAATCCTTGGCCCTCCATCCTTAGATAGtccggcaattAAAAAAGCGGCTAACCACGCCGCTTTTTTtacgtctgcaTCATAGGCAATACGATCGCATGTCC | ([Chen et al, 2013](#_ENREF_4)) |
| DT16 | Terminator | CTCGGTACCAAATTCCAGAAAAGAGGCCTCCCGAAAGGGGGGCCTTTTTTCGTTTTGGTCCTCCTTGGCCCTCCATCCTTAGATAGTCCGGCAATTAAAAAAGCGGCTAACCACGCCGCTTTTTTTACGTCTGCATCATAGGCAATACGATCGCATGTCC | ([Chen et al, 2013](#_ENREF_4)) |
| DT61 | Terminator | GGACCAAAACGAAAAAAGACGCTTTTCAGCGTCTTATTGTTCGTCTTTGGTACCGAGTGTTGTGCTGGGAATAGTGTAGTCATGCAGATAACAAAAAACCCCGCCGGAGCGAGGTTTCGTCAGTCGCCT | ([Chen et al, 2013](#_ENREF_4)) |
| DT11 | Terminator | AACGCATGAGAAAGCCCCCGGAAGATCACCTTCCGGGGGCTTTTTTATTGCGCTCCTTGGCCCTCCATCCTTAGATAGCTCGGTACCAAATTCCAGAAAAGAGGCCTCCCGAAAGGGGGGCCTTTTTTCGTTTTGGTCCTCATAGGCAATACGATCGCATGTCC | ([Nielsen et al, 2016](#_ENREF_19)) |
| TrrnB | Terminator | GAAGCTTGGGCCCGAACAAAAACTCATCTCAGAAGAGGATCTGAATAGCGCCGTCGACCATCATCATCATCATCATTGAGTTTAAACGGTCTCCAGCTTGGCTGTTTTGGCGGATGAGAGAAGATTTTCAGCCTGATACAGATTAAATCAGAACGCAGAAGCGGTCTGATAAAACAGAATTTGCCTGGCGGCAGTAGCGCGGTGGTCCCACCTGACCCCATGCCGAACTCAGAAGTGAAACGCCGTAGCGCCGATGGTAGTGTGGGGTCTCCCCATGCGAGAGTAGGGAACTGCCAGGCATCAAATAAAACGAAAGGCTCAGTCGAAAGACTGGGCCTTTCGTTTTATCTGTTGTTTGTCGGTGAACT | ([Chen et al, 2013](#_ENREF_4)) |
| TihfA | Terminator | AGTGAAAAGAAAAAAGGCCGCAGAGCGGCCTTTTTAGTTAGATC | ([Chen et al, 2013](#_ENREF_4)) |
| Tind | Terminator | TAATTGGTAACGAATCAGACAATTGACGGCTCGAGGGAGTAGCATAGGGTTTGCAGAATCCCTGCTTCGTCCATTTGACAGGCACATTATGCATCGATGATAAGCTGTCAAACATGAGCAGATCCTCTACGCCGGACGCATCGTGGCCGGCATCACCGGCGCCACAGGTGCGGTTGCTGGCGCCTATATCGCCGACATCACCGATGGGGAAGATCGGGCTCGCCACTTCGGGCTCATGAGCAAATATTTTATCTG | ([Nielsen & Voigt, 2014](#_ENREF_20)) |
| Context1 | Random spacer | AGAATCACCGACGAACACAGGATACGACAAGGCACTGGGCAACGAGAACACATAACACTAGTCACCCGCGATCAATTAGACCAAAAAGCATTCGCTAGACCACAGCG | This work |
| Context2 | Random spacer | TGCATACAAAAGCCACCTACAAAGCGACAAACGCGCCAGAGCCCAGACCAACAAATGAATATGACAGACACCTCCCCCGTGGCAAAGAAAATAGGGAACCGGAGAAT | This work |
| Context3 | Random spacer | GCGTCAAATACTGAAGCCGCGAGATGGACGCCTTATAAAGTGCCAACCGTAATACACACGGCAGAGACAAACAAAGATGTAGACGCAGCTCCTGCCCTCAAACTACT | This work |

*The first 6 bp of HH2 and HH3 ribozyme sequences (NNNNNN) are the reverse complement of the first 6 bp of the targeting sequence that follows the central ribozyme sequence.

**II. References**

(2016) Registry of Standard Biological Parts. Biobricks Foundation, Vol. 2016.

Aiba H (1985) Transcription of the Escherichia coli adenylate cyclase gene is negatively regulated by cAMP-cAMP receptor protein. *J Biol Chem* **260:** 3063-3070

Cameron DE, Collins JJ (2014) Tunable protein degradation in bacteria. *Nat Biotechnol* **32:** 1276-1281

Chen YJ, Liu P, Nielsen AA, Brophy JA, Clancy K, Peterson T, Voigt CA (2013) Characterization of 582 natural and synthetic terminators and quantification of their design constraints. *Nat Methods* **10:** 659-664

Collado-Vides J, Magasanik B, Gralla JD (1991) Control site location and transcriptional regulation in Escherichia coli. *Microbiol Rev* **55:** 371-394

Datsenko KA, Wanner BL (2000) One-step inactivation of chromosomal genes in Escherichia coli K-12 using PCR products. *Proc Natl Acad Sci U S A* **97:** 6640-6645

Farmer WR, Liao JC (2001) Acetate-inducible protein overexpression from the glnAp2 promoter of Escherichia coli. *Biotechnol Bioeng* **75:** 504-509

Fernandez-Rodriguez J, Voigt CA (2016) Post-translational control of genetic circuits using Potyvirus proteases. *Nucleic acids research* **44:** 6493-6502

Garcia JR, Cha HJ, Rao G, Marten MR, Bentley WE (2009) Microbial nar-GFP cell sensors reveal oxygen limitations in highly agitated and aerated laboratory-scale fermentors. *Microb Cell Fact* **8:** 6

Ghodasara A, Voigt CA (2017) Balancing gene expression without library construction via a reusable sRNA pool. *Nucleic acids research* **45:** 8116-8127

Hendrickson W, Stoner C, Schleif R (1990) Characterization of the Escherichia coli araFGH and araJ promoters. *Journal of molecular biology* **215:** 497-510

Huang L, Tsui P, Freundlich M (1992) Positive and negative control of ompB transcription in Escherichia coli by cyclic AMP and the cyclic AMP receptor protein. *J Bacteriol* **174:** 664-670

Kang Y, Weber KD, Qiu Y, Kiley PJ, Blattner FR (2005) Genome-wide expression analysis indicates that FNR of Escherichia coli K-12 regulates a large number of genes of unknown function. *J Bacteriol* **187:** 1135-1160

Lewis DE, Adhya S (2015) Molecular Mechanisms of Transcription Initiation at gal Promoters and their Multi-Level Regulation by GalR, CRP and DNA Loop. *Biomolecules* **5:** 2782-2807

Liu D, Zhang F (2018) Metabolic Feedback Circuits Provide Rapid Control of Metabolite Dynamics. *ACS Synth Biol* **7:** 347-356

Lou C, Stanton B, Chen YJ, Munsky B, Voigt CA (2012) Ribozyme-based insulator parts buffer synthetic circuits from genetic context. *Nat Biotechnol* **30:** 1137-1142

Moon TS, Lou C, Tamsir A, Stanton BC, Voigt CA (2012) Genetic programs constructed from layered logic gates in single cells. *Nature* **491:** 249-253

Neumann P, Weidner A, Pech A, Stubbs MT, Tittmann K (2008) Structural basis for membrane binding and catalytic activation of the peripheral membrane enzyme pyruvate oxidase from Escherichia coli. *Proc Natl Acad Sci U S A* **105:** 17390-17395

Nielsen AA, Der BS, Shin J, Vaidyanathan P, Paralanov V, Strychalski EA, Ross D, Densmore D, Voigt CA (2016) Genetic circuit design automation. *Science* **352:** aac7341

Nielsen AA, Voigt CA (2014) Multi-input CRISPR/Cas genetic circuits that interface host regulatory networks. *Mol Syst Biol* **10:** 763

Oxer MD, Bentley CM, Doyle JG, Peakman TC, Charles IG, Makoff AJ (1991) High level heterologous expression in E. coli using the anaerobically-activated nirB promoter. *Nucleic acids research* **19:** 2889-2892

Qi LS, Larson MH, Gilbert LA, Doudna JA, Weissman JS, Arkin AP, Lim WA (2013) Repurposing CRISPR as an RNA-guided platform for sequence-specific control of gene expression. *Cell* **152:** 1173-1183

Raghavan R, Sage A, Ochman H (2011) Genome-wide identification of transcription start sites yields a novel thermosensing RNA and new cyclic AMP receptor protein-regulated genes in Escherichia coli. *J Bacteriol* **193:** 2871-2874

Sa JH, Namgung MA, Lim CJ, Fuchs JA (1997) Expression of the Escherichia coli thioredoxin gene is negatively regulated by cyclic AMP. *Biochem Biophys Res Commun* **234:** 564-567

Salmon K, Hung SP, Mekjian K, Baldi P, Hatfield GW, Gunsalus RP (2003) Global gene expression profiling in Escherichia coli K12. The effects of oxygen availability and FNR. *J Biol Chem* **278:** 29837-29855

Segall-Shapiro TH, Meyer AJ, Ellington AD, Sontag ED, Voigt CA (2014) A 'resource allocator' for transcription based on a highly fragmented T7 RNA polymerase. *Mol Syst Biol* **10:** 742

Stanton BC, Nielsen AA, Tamsir A, Clancy K, Peterson T, Voigt CA (2014a) Genomic mining of prokaryotic repressors for orthogonal logic gates. *Nat Chem Biol* **10:** 99-105

Stanton BC, Siciliano V, Ghodasara A, Wroblewska L, Clancy K, Trefzer AC, Chesnut JD, Weiss R, Voigt CA (2014b) Systematic transfer of prokaryotic sensors and circuits to mammalian cells. *ACS Synth Biol* **3:** 880-891
